# Supplementary material for: Prospective observational study of prevalence, assessment and treatment of pancreatic exocrine insufficiency in patients with inoperable pancreatic malignancy (PANcreatic cancer Dietary Assessment (PanDA): a study protocol
Source: BMJ Open. 2021 May 13;11(5):e042067. doi: 10.1136/bmjopen-2020-042067 (PMC8126274; doi:10.1136/bmjopen-2020-042067)
Supplement: Author's manuscript [file bmjopen-2020-042067.draft_revisions.pdf]

BMJ Open

BMJ Open is committed to open peer review. As part of this commitment we make the peer review history of every article we publish publicly available.

When an article is published we post the peer reviewers' comments and the authors' responses online. We also post the versions of the paper that were used during peer review. These are the versions that the peer review comments apply to.

The versions of the paper that follow are the versions that were submitted during the peer review process. They are not the versions of record or the final published versions. They should not be cited or distributed as the published version of this manuscript.

BMJ Open is an open access journal and the full, final, typeset and author-corrected version of record of the manuscript is available on our site with no access controls, subscription charges or pay-per-view fees (http://bmjopen.bmj.com).

If you have any questions on BMJ Open's open peer review process please email info.bmjopen@bmj.com

BMJ Open

Prospective observational study of prevalence, assessment and treatment of pancreatic exocrine insufficiency in patients with inoperable pancreatic malignancies (PANcreatic cancer Dietary Assessment - PanDA): a study protocol

Journal:	*BMJ Open*
Manuscript ID	bmjopen-2020-042067
Article Type:	Protocol
Date Submitted by the Author:	24-Jun-2020
Complete List of Authors:	Carnie, Lindsay; The Christie NHS Foundation Trust, Nutrition & Dietetics<br>Lamarca, Angela; The Christie NHS Foundation Trust, Medical Oncology Department; The University of Manchester, Division of Cancer Sciences<br>Vaughan, Kate; The Christie NHS Foundation Trust, Medical Oncology; The University of Manchester, Division of Cancer Sciences<br>Kapacee, Zainul Abedin ; The Christie NHS Foundation Trust, Medical Oncology<br>Mccallum, Lynne; Pancreatic Cancer UK<br>Backen, Alison; The Christie NHS Foundation Trust; The University of Manchester, Division of Cancer Sciences<br>Barriuso, Jorge; The Christie NHS Foundation Trust, Medical Oncology; The University of Manchester, Division of Cancer Sciences<br>McNamara, Mairéad ; The Christie NHS Foundation Trust; The University of Manchester, Division of Cancer Sciences<br>Hubner, R; The Christie NHS Foundation Trust, Medical Oncology; The University of Manchester, Division of Cancer Sciences<br>Abraham†, Marc; The Christie NHS Foundation Trust, Nutrition & Dietetics<br>Valle, Juan; The Christie NHS Foundation Trust, Medical Oncology; The University of Manchester, Division of Cancer Sciences
Keywords:	Pancreatic disease < GASTROENTEROLOGY, Gastrointestinal tumours < ONCOLOGY, Nutritional support < ONCOLOGY

SCHOLARONE™
Manuscripts

I, the Submitting Author has the right to grant and does grant on behalf of all authors of the Work (as defined in the below author licence), an exclusive licence and/or a non-exclusive licence for contributions from authors who are: i) UK Crown employees; ii) where BMJ has agreed a CC-BY licence shall apply, and/or iii) in accordance with the terms applicable for US Federal Government officers or employees acting as part of their official duties; on a worldwide, perpetual, irrevocable, royalty-free basis to BMJ Publishing Group Ltd ("BMJ") its licensees and where the relevant Journal is co-owned by BMJ to the co-owners of the Journal, to publish the Work in this journal and any other BMJ products and to exploit all rights, as set out in our licence.

The Submitting Author accepts and understands that any supply made under these terms is made by BMJ to the Submitting Author unless you are acting as an employee on behalf of your employer or a postgraduate student of an affiliated institution which is paying any applicable article publishing charge ("APC") for Open Access articles. Where the Submitting Author wishes to make the Work available on an Open Access basis (and intends to pay the relevant APC), the terms of reuse of such Open Access shall be governed by a Creative Commons licence – details of these licences and which Creative Commons licence will apply to this Work are set out in our licence referred to above.

Other than as permitted in any relevant BMJ Author's Self Archiving Policies, I confirm this Work has not been accepted for publication elsewhere, is not being considered for publication elsewhere and does not duplicate material already published. I confirm all authors consent to publication of this Work and authorise the granting of this licence.

For peer review only

Contents

Title Page 3

Abstract 4

Strengths and limitations of the study 5

Introduction 6

The pancreas 6

Pancreatic cancer | The importance of being fit for treatment 6

Pancreatic exocrine insufficiency | Causing malnutrition in patients with pancreatic cancer 7

Diagnosis of PEI in patients with pancreatic malignancy 8

Treatment of PEI and its impact on Quality of Life and survival 9

Aim 10

Study design 10

Study objectives and patient eligibility 10

Demographic cohort 10

Diagnostic cohort 12

Follow-up cohort 12

Clinical assessments 13

Demographic cohort 14

Screening and Visit 1 14

Follow-up visits 14

Diagnostic cohort 15

Screening and Visit 1 15

For peer review only - http://bmjopen.bmj.com/site/about/guidelines.xhtml

Follow-up visits 15
Follow-up cohort 15
Screening and Visit 1 16
Visit 2 (within two weeks) 16
Week 4-6 from study entry 16
Follow-up visits 16
Statistical analysis 17
Sample size 17
Study end-points 18
Demographic cohort; 18
Diagnostic cohort; 18
Follow-up cohort; 18
Data analysis 18
Ethics and dissemination 19
Author's contributions 20
Funding statement 20
Ethical approval 21
References 22

For peer review only - http://bmjopen.bmj.com/site/about/guidelines.xhtml

Title Page

Prospective observational study of prevalence, assessment and treatment of pancreatic exocrine insufficiency in patients with inoperable pancreatic malignancies (PANcreatic cancer Dietary Assessment - PanDA): a study protocol

List of authors

Lindsay Carnie [(1)], Angela Lamarca [(2, 3)], Kate Vaughan [(2, 3)], Zainul Abedin Kapacee [(2)], Lynne McCallum [(4)], Alison Backen [(2,3)], Jorge Barriuso[(2,3)], Mairéad G McNamara[(2, 3)], Richard A Hubner[(2, 3)], Marc Abraham [(1)], Juan W Valle [(2, 3)]

List of affiliations

1. Nutrition & Dietetics, The Christie NHS Foundation Trust, Manchester, UK
2. Medical Oncology Department, The Christie NHS Foundation Trust, Manchester, UK
3. Division of Cancer Sciences, University of Manchester, UK
4. Pancreatic Cancer UK, London, UK

Corresponding author: Prof Juan W Valle, Medical Oncology Department, The Christie NHS Foundation Trust, Wilmslow Road, Manchester, M20 4BX, United Kingdom. E-mail: juan.valle@christie.nhs.uk

Key words: pancreatic ductal adenocarcinoma, pancreatic neuroendocrine tumours, dietitian, malnutrition, pancreatic exocrine insufficiency, pancreatic enzyme replacement therapy, breath test, faecal elastase

Word Count: 3733 words (excluding title page, abstract, references, figures and tables)

For peer review only - http://bmjopen.bmj.com/site/about/guidelines.xhtml

For peer review only

Abstract

Introduction

Pancreatic exocrine insufficiency (PEI) in patients with pancreatic malignancies is well documented in the literature and is known to negatively impact on overall survival and quality of life. A lack of consensus opinion remains on the optimal diagnostic test that can be adapted for use in a clinical setting for this cohort of patients. This study aims to better understand the prevalence of PEI and the most suitable diagnostic techniques in patients with advanced pancreatic malignancies.

Methods and analysis

This prospective observational study, will be carried out in patients with pancreatic malignancies (including adenocarcinoma and neuroendocrine neoplasms). Consecutive patients with inoperable pancreatic malignancies referred for consideration of first-line chemotherapy will be considered for eligibility. The study comprises of three cohorts: demographic cohort (primary objective to prospectively investigate the prevalence of PEI in patients with inoperable pancreatic malignancies) ; sample size 50, diagnostic cohort (primary objective to design and evaluate an optimal diagnostic panel to detect PEI in patients with inoperable pancreatic malignancies); sample size 25 and follow-up cohort (primary objective to prospectively evaluate the proposed PEI diagnostic panel in a cohort of patients with inoperable pancreatic malignancies); sample size 50. The following is a summary of the protocol and methodology.

Ethics and dissemination

Full ethical approval has been granted by the North West Greater Manchester East Research and Ethics Committee, reference: 17/NW/0597. This manuscript reflects the latest protocol v.8 approved 21st April, 2020.

For peer review only - http://bmjopen.bmj.com/site/about/guidelines.xhtml

Trial registration number

IRAS project ID: 194255.

Clinicaltrials.gov ID: NCT03616431

Strengths and limitations of the study

- This prospective study is a first-of-its-kind in patients with inoperable pancreatic malignancies
- A prospective assessment of the prevalence PEI-related symptoms and the impact of pancreatic enzyme replacement therapy will inform treatment and management of future patients
- The ^{13}C-mixed triglyceride breath test has not previously been tested for acceptability in patients with inoperable pancreatic malignancies
- A high attrition rate is expected, due to the nature of inoperable pancreatic adenocarcinoma with a median survival of <6 months

For peer review only - http://bmjopen.bmj.com/site/about/guidelines.xhtml

Introduction

The pancreas

The pancreas has two main functions; producing enzymes to digest protein, fat and carbohydrates into smaller molecules that the body can absorb, and producing hormones that regulate metabolism (including the regulation of blood sugar levels (insulin and glucagon) and global regulation of other hormones). [1]

Pancreatic cancer | The importance of being fit for treatment

Pancreatic cancer (adenocarcinoma) is known to have a poor prognosis with a very low cure rate; most patients diagnosed will die of the disease. In 2014, around 41,000 pancreatic cancer-related deaths occurred in Europe.[2]

The physical location of the tumour can prevent the digestive regulatory functions of the pancreas, causing the systemic symptoms that the majority of patients present with. Symptoms include anorexia (83%), asthenia (86%) and weight loss (85%). [3] Symptoms can impact on Quality of Life (QoL), nutritional status and performance status (PS), which subsequently may preclude active treatment options such as chemotherapy.[4]

Only approximately 20% of patients are suitable for surgery at diagnosis; these patients undergo pancreatic resection followed by adjuvant chemotherapy with fluoropyrimidine-based or gemcitabine-based treatment.[5] [6] [7] A good nutritional status, prior to adjuvant chemotherapy increases the likelihood of a patient completing chemotherapy, which in turn impacts on survival. [8]

Most patients (80%) present with advanced disease and are unsuitable for surgery. Instead they will receive palliative chemotherapy, aiming to improve QoL and prolong overall survival (OS). Single-agent gemcitabine has long been considered standard of care in patients with a poorer performance

For peer review only

For peer review only - http://bmjopen.bmj.com/site/about/guidelines.xhtml

status, providing a median OS of 6 months. [9] Recent chemotherapy combinations show improved results, reaching a median OS of 8.5 months (nab-paclitaxel/gemcitabine), [10] and 11.1 months (FOLFIRINOX; a 5-fluorouracil, oxaliplatin and irinotecan combination). [11]

A retrospective analysis of patients with advanced pancreatic cancer referred to The Christie NHS Foundation Trust found around 40% were not fit for active treatment due to poor baseline PS as per The Eastern Cooperative Oncology Group – PS (ECOG-PS) definition. [12]

The scenario for patients diagnosed with pancreatic neuroendocrine tumours (PanNETs) differs significantly. Prognosis is measured in term of years, with an estimated median OS of 3.6 years [13] and multiple options of systemic therapy are currently available.[14] The prevalence of PanNETs is rare, with an estimated incidence of 0.8 per 100,000. [13] Whilst the prognosis of these patients is better, this longer survival time means that identified and minimising the impact of nutritional deficiencies and issues is of particular importance. [15]

Pancreatic exocrine insufficiency | Causing malnutrition in patients with pancreatic cancer

Pancreatic exocrine insufficiency (PEI) is defined as "a reduction in pancreatic enzyme activity in the intestinal lumen to a level below the threshold required to maintain normal digestion".[16]

A high prevalence of PEI has been described in patients with resected (>80%) [17] or advanced-disease (92%) [18] in prospective series, and this negatively impacts on QoL. [19] Different mechanisms have been postulated for the development of PEI, including loss of functioning pancreatic parenchyma (by tumour infiltration or resection or concurrent/prior pancreatitis) and/or pancreatic duct obstruction. PEI, leading to maldigestion, steatorrhoea and malnutrition, has been proposed as a leading cause for the high number of patients with pancreatic malignancies being unfit for active treatment. [20]

For peer review only - http://bmjopen.bmj.com/site/about/guidelines.xhtml

Whilst healthcare professionals seem aware of the importance of diagnosing and treating PEI in patients after pancreatic resection, it is often overlooked in patients with advanced disease. This under-recognition and under-treatment of PEI in patients with advanced disease is an ongoing issue, requiring urgent action. [21]

Weight loss is a poor prognostic factor in patients with both resectable and advanced pancreatic malignancies. [22,23] However, little published information exists on the extent of nutritionally-mediated weight loss, how this relates to the cancer, and how much could be mitigated with pro-active pancreatic enzyme replacement therapy (PERT).

Diagnosis of PEI in patients with pancreatic malignancy

Waiting for symptom development, including steatorrhoea (defined as excess fat in faeces that appears when 90% of pancreatic function is lost) delays the diagnosis of PEI and negatively impacts on nutrition and QoL. [24] Early assessment of exocrine function is fundamental, and should be considered in all patients diagnosed with pancreatic disorders, including cancer. [25]

Diagnosing PEI in patients with pancreatic malignancy can be difficult, and a lack of consensus remains for the optimal assessment method. Whilst three-day faecal fat quantification is 'gold-standard' for diagnosing PEI, its use in clinical practice is challenging. [16] The secretin test is invasive and has potential for clinical complications, reducing its appeal. [26] Measurable reduction of pancreatic parenchymal thickness in imaging correlates with changes assessed using a ^{13}C-mixed triglyceride breath test (^{13}C-MTBT), with good sensitivity and specificity after pancreatic resection. [27] This has become the new 'standard', replacing the three-day faecal fat test. The use of current diagnostic techniques such as faecal elastase-1 (FE-1), [28] (postulated to be more useful in patients who have not undergone resection), the ^{13}C-MTBT [29] and a nutritional panel of blood-based markers warrant further investigation to clarify their use. [30]

For peer review only - http://bmjopen.bmj.com/site/about/guidelines.xhtml

In summary | The optimal diagnostic method for PEI in patients with pancreatic malignancy remains undefined; ^{13}C-MTBT is considered 'gold-standard' but is challenging to apply in daily clinical settings. This study aims to design the most appropriate and least-invasive diagnostic panel, with ^{13}C-MTBT as the comparator for patients diagnosed with pancreatic malignancies (including both adenocarcinoma and neuroendocrine tumours).

Treatment of PEI and its impact on Quality of Life and survival

Guidelines for the management of PEI exist,[31–33] and two publications support using high-dose PERT to mimic the physiological situation, to normalise nutritional status.[29,34] Using a proton pump inhibitor to increase gastric pH, enhancing the efficacy of PERT (by reducing gastric acid-induced enzymatic degradation) in selected patients has also been demonstrated. [35]

At The Christie NHS Foundation Trust, 183 patients with pancreatic malignancies were retrospectively analysed and it was demonstrated that patients receiving nutritional intervention (PERT, nutritional supplements or dietitian support) seemed to receive more chemotherapy and had a longer OS [10.2 months (95%CI 7.5-13.3) vs 6.9 months (95%CI 5.5-9.9); HR 0.6 (95%CI 0.4-0.9); p-value 0.015], when adjusted for other variables in the multivariable analysis (type of pancreatic cancer, stage at diagnosis, ECOG-PS and chemotherapy treatment)]. [12] This study also confirmed that PEI is under-recognised and under-treated in patients with advanced disease. Since this was a retrospective study, it is subject to selection and survival bias. Therefore, whilst results are encouraging, prospective studies are required to evaluate the impact of dietetic intervention (including PERT) on QoL, exposure to anti-cancer treatment, symptom control and outcome.

In summary | Dietetic intervention, early diagnosis and management of PEI could impact patients' OS. This study aims to prospectively assess the impact of such interventions in patients with pancreatic malignancies.

For peer review only - http://bmjopen.bmj.com/site/about/guidelines.xhtml

Aim

This prospective observational study aims to evaluate;

- The prevalence of PEI in patients with pancreatic ductal adenocarcinoma and PanNETs (hence force termed pancreatic malignancies).
- The most appropriate diagnostic strategy.
- The impact of adequate diagnosis and treatment of PEI on patient treatment and outcomes.

Study design

The study will be conducted in two steps, as summarised in **Figure 1**.

Step-1 | A prospective cross-sectional assessment of the prevalence of PEI-related symptoms in patients with pancreatic malignancy (this will be termed 'the **Demographic cohort**'). A separate cohort of patients will be tested to elucidate the most efficient diagnostic panel for PEI in pancreatic malignancy (this will be termed 'the **Diagnostic cohort**').

Step-2 | A prospective longitudinal validation of the diagnostic panel designed and tested in Step-1 and evaluation of dietitian intervention (including PERT) and its impact on weight loss, symptom evolution, chemotherapy dose-intensity, QoL and OS (this will be termed 'the **Follow-up cohort**').

Study objectives and patient eligibility

A summary of study objectives are provided in **Figure 2**.

Demographic cohort

The primary objective is to prospectively investigate the prevalence of PEI in patients with inoperable pancreatic malignancies. Prevalence will be determined by the presence of symptoms

For peer review only - http://bmjopen.bmj.com/site/about/guidelines.xhtml

deemed in-keeping with PEI by the research dietitian; alongside the absence of another causes for symptoms or standard diagnostic techniques (FE-1).

Secondary objectives include, at baseline oncological appointment;

- To assess the proportion of patients receiving PERT
- To evaluate nutritional status (using a panel of blood tests (including nutritional parameters), weight, Body Mass Index [BMI], Mid-Upper Arm Circumference [MUAC] (reflects both fat mass and fat-free mass), handgrip strength (measures upper body function) and Stair Climb test [SC-test] (to calculate stair climb power[36,37]))
- To evaluate anorexia, using the Functional Assessment of Anorexia/Cachexia Therapy questionnaire (FAACT–A/CS) and Visual Analogue Scale (VAS) [38,39]

Eligible patients for the demographic cohort are those who have biopsy-proven or clinically-suspected (by specialist multidisciplinary team (MDT) meeting) inoperable (locally-advanced or metastatic) pancreatic ductal adenocarcinoma (and variants) or PanNET. There is no minimal time-frame for patients to have been diagnosed with cancer. Patients must be ≥18 years and able to provide written, informed consent and are being considered for first-line chemotherapy. Patients with PanNET may have received previous systemic treatment, but cannot be on active treatment.

Patients are deemed ineligible if they have had previous gastric, duodenal or pancreatic resections, if they have an intolerance/aversion to pork-containing products for religious or personal reasons. Additionally, patients are ineligible if they have comorbidities that increase the probability of PEI, including but not limited to: chronic pancreatitis, [25] cystic fibrosis,[40] coeliac disease,[41] inflammatory bowel disease,[42,43] diarrhoea-dominant irritable bowel syndrome,[44] diabetes diagnosed > 5 years ago. [45–47]

For peer review only - http://bmjopen.bmj.com/site/about/guidelines.xhtml

Diagnostic cohort

The primary objective is to design and evaluate an optimal diagnostic panel to detect PEI in patients with inoperable pancreatic malignancies.

In addition to the secondary objectives of the Demographic cohort;

- To assess the feasibility and acceptability (using a specifically designed "Acceptability Questionnaire") of the ^{13}C-MTBT and the FE-1 test

Eligible patients for the diagnostic cohort are those who fulfil the eligibility criteria for the demographic cohort. In addition, patients with potentially operable disease but who have not undergone surgery for whatever reason (i.e. co-morbidities) would be eligible if all other eligibility criteria are met. Additionally, patients diagnosed with adenocarcinoma (and variants) will be allowed to have received previous systemic treatment but will require to be off active treatment for a minimum of 3 months to be included in this cohort.

In addition to the exclusion criteria for the demographic cohort, patients must not be allergic to metoclopramide, a prokinetic used in the ^{13}C-MTBT.

Follow-up cohort

The primary objective is to prospectively evaluate the proposed PEI diagnostic panel in a cohort of patients with inoperable pancreatic malignancies.

Secondary objectives include;

- To evaluate the feasibility of applying the designed diagnostic panel in clinical practice and to assess the patient acceptability of these investigations

For peer review only - http://bmjopen.bmj.com/site/about/guidelines.xhtml

- To quantify changes to nutritional status (BMI, weight, MUAC, handgrip and SC-test), evaluate symptoms, and extent of diagnostic panel normalisation, anorexia (FAACT–A/CS (with VAS) and QoL at 6 weeks, 3 months and 6 months after recruitment and dietetic input
- To evaluate PERT compliance and toxicity
- To assess patient perceptions of the dietetic care provided
- To evaluate the impact of dietetic intervention on OS, anti-cancer therapy starting rate & anti-cancer therapy dose intensity
- Exploratory radiological surrogates (i.e. intra-abdominal fat) from standard of care Computed Tomography (CT) scans and its correlation with nutritional assessment may be investigated
- To evaluate the median dose intensity of the received anti-cancer therapy and the correlation between radiological findings and nutritional status measurements [48]

Eligible patients for the follow-up cohort are those fulfilling the eligibility criteria for the diagnostic cohort and if further follow-up at The Christie NHS Foundation Trust is planned.

Clinical assessments

Consecutive patients with inoperable pancreatic malignancies referred for consideration of first-line systemic therapy will be considered for eligibility. Eligible patients will be provided with verbal and written study information and given sufficient time to consider participation.

Clinical assessments will be undertaken as per **Figure 3**. All consenting patients will undergo a prospective assessment of nutritional status, will be screened for PEI and will receive tailored advice by the research dietitian in established oncology clinics.

For peer review only - http://bmjopen.bmj.com/site/about/guidelines.xhtml

Demographic cohort

Screening and Visit 1

At baseline, patients will be screened against inclusion criteria. Written, informed consent must be granted before patients are registered. If appropriate, the baseline assessment can be performed on the same day as screening.

Assessments undertaken are as follows (**Figure 3**):

- Physical examination; vital signs, height, weight, BMI, MUAC, handgrip strength, SC-test and FAACT-A/CS (with VAS)
- Baseline symptoms (PEI-related) and graded as per Common Terminology Criteria for Adverse Events version 4.03 (CTCAE v 4.03) [49]
- PEI-relevant concomitant medications
- ECOG-PS
- Blood collection for nutritional panel
- Dietitian assessment and counselling (PERT will be commenced, if required)
- PERT treatment and toxicity assessment (if patient on PERT)

Follow-up visits

No follow-up visits will be required. Beyond study participation, dietetic input will be provided as per standard of care outside the context of this study. Information on subsequent chemotherapy treatment (starting rate and dose intensity) and survival outcomes will be collected.

For peer review only

For peer review only - http://bmjopen.bmj.com/site/about/guidelines.xhtml

Diagnostic cohort

Screening and Visit 1

The baseline assessment will be completed as per the “Demographic cohort”.

Visit 2 (prior to starting systemic treatments)

The following assessments will be performed:

- Weight
- FE-1 test (container provided at baseline visit and returned at visit 2)
- ^{13}C-MTBT (takes around six hours to complete, including administering bread spread with ^{13}C butter and subsequent collection of the patient’s breath in small breath bags at timed intervals, which will be analysed for ^{13}C quantity)
- Acceptability questionnaire for FE-1 test and ^{13}C-MTBT (all patients) to provide opinions on the burden that these extra tests may add

Follow-up visits

No follow-up visits will be required. Patients attending clinic for further follow-up/treatment, will have further dietetic input, as required, outside of the context of this study. Information on the subsequent chemotherapy (starting rate and dose intensity) and survival outcomes will be collected.

Follow-up cohort

Prior to opening recruitment to this cohort, data from the Demographic and Diagnostic cohorts will be analysed, which will dictate the most informative diagnostic panel devised to be used in the Follow-up cohort.

For peer review only - http://bmjopen.bmj.com/site/about/guidelines.xhtml

Screening and Visit 1

Screening will be completed as per the Demographic and Diagnostic cohorts.

In addition, further assessments (**Figure 3**) include:

- QoL questionnaires (QLQ-C30 (all patients) and either QLQ-PAN26 (pancreatic ductal adenocarcinoma) or QLQ NET-21 (PanNET))
- A symptom and PERT diary for data collection will be provided

Visit 2 (within two weeks)

- Designed PEI diagnostic panel (from all the potential combinations of FE-1 test, symptom assessment, nutritional assessment [weight, BMI, MUAC, handgrip strength, SC-test, FAACT–A/CS (with VAS) and nutritional blood panel]), as per findings from the Diagnostic cohort)
- "Acceptability Questionnaire" regarding the burden that this diagnostic panel added

Week 4-6 from study entry

- "Feedback questionnaire" regarding perception of dietetic input (all patients) (posted to the patient and returned using a provided stamped-addressed envelope)

Follow-up visits

At six weeks, three months and six months after recruitment;

- Weight, BMI, MUAC, handgrip strength, SC-test and FAACT-A/CS (with VAS)
- Physical examination (symptom directed), including vital signs (if appropriate)
- ECOG-PS
- Dietitian assessment and counselling, nutrition support advice (diet, nutritional supplements, etc.) and PERT, as required

For peer review only

- PERT treatment review and toxicity assessment (if taking PERT)
- Symptom and PERT diary collection
- QoL questionnaires repeated as per visit 1
- Survival and chemotherapy treatment monitoring (retrospectively)

Patients attending clinic for further follow-up/treatment will be provided with dietetic input, as required, outside of the context of this study.

Statistical analysis

Sample size

No formal sample size calculation was performed. Instead, a realistic estimation of the number of patients possible to recruit was made using established referral rates and the length of time this study will recruit for. A high drop-out rate was expected due to the poor outcomes of patients with pancreatic cancer. Therefore, sufficient patients will be recruited to ensure the planned number of 'evaluable patients' for each cohort is reached. These are defined as;

Demographic cohort: up to 50 eligible patients completing the assessment required in "Visit 1 "

Diagnostic cohort: up to 25 eligible patients willing (at time of consent) to complete breath test and other cohort-dependent examinations

Follow-up cohort: up to 50 eligible patients completing assessments up to and including "Follow-up visit week 6"

Handgrip strength measurements contribute to the statistical calculation; In order to gather supporting data, a non-selected sample of 12 patients with pancreatic ductal adenocarcinoma performed the handgrip test as per standard of care assessment at The Christie (mean percentile was 75; standard deviation of 16). Using these data, the Follow-up cohort (50 patients with handgrip

For peer review only

assessment at baseline and at 6 weeks) will have a power of 0.75 to show an improvement from 75 to 82 (seven point improvement). This is assuming an alpha error of 0.15 and same standard deviation in both baseline and 6-week assessment handgrip results (standard deviation of 16). This is supporting evidence that the sample size for this study will be able to provide meaningful and robust results.

Study end-points

The primary end-points of the three cohorts are;

Demographic cohort;

- Proportion of patients with symptoms/findings in keeping with a PEI diagnosis

Diagnostic cohort;

- Odds ratio for prediction of diagnosis of PEI (measured by ^{13}C-MTBT) of the most accurate diagnostic panel (designed from all potential combinations of FE-1 test, symptom assessment, nutritional assessment [weight, BMI, MUAC, handgrip strength, SC-test, FAACT–A/CS (with VAS) and nutritional blood panel])

Follow-up cohort;

- Rate of PEI diagnosis according to the designed diagnostic panel (diagnostic cohort)

Data analysis

Frequency tables for all categorical variables, arranged by category, will be produced for comparison. Continuous variables (age, weight, BMI, MUAC, handgrip strength, SC-test and FAACT–A/CS (with VAS) will be presented, using the median and range (minimum, maximum) or mean (variance), depending on whether data distribution appears symmetrical.

For peer review only

For exploratory analyses, means will be compared using either Student T test (if parametric validity conditions are fulfilled) or non-parametric Wilcoxon-Mann-Whitney tests. Proportions will be compared using either Chi-squared statistics or Fisher exact test, as appropriate. Toxicity data will be tabulated. The worst toxicity grade over all cycles according to the CTCAE v 4.03 [49] will be reported.

Median survival will be calculated using the Kaplan-Meier estimator technique. Median OS will be displayed with the 95% confidence interval. For comparison of survival curves, Log-rank test will be applied. Multivariable analyses will also be performed (Cox Regression).

Analysis of data collected from the Demographic and Diagnostic cohorts will be undertaken to devise the optimal diagnostic panel, using ^{13}C-MTBT as a reference to diagnose PEI. Results from the breath test will be reported as a dichotomised variable (normal or abnormal).

Logistic regression will be performed, aiming to choose the most informative, but simplest panel of tests, to predict PEI as the ^{13}C-MTBT has done.

Individually measured blood parameters, together with other calculated scores (such as, but not limited to the "prognostic nutritional index" (combining lymphocytes and albumin)) will be included in such analysis, if required.

Further analysis upon the completion of the Follow-up cohort will evaluate the panel's accuracy and acceptability for use in clinical practice.

Ethics and dissemination

The study will be conducted according to the principles of Good Clinical Practice (GCP), General Data Protection Regulation (GDPR) and data protection Act 2018 for Health and Care Research.

The sponsor and study team will ensure approval of the study protocol, participant information sheets, consent forms, letters to General Practitioners and supporting documents by the appropriate

For peer review only

For peer review only - http://bmjopen.bmj.com/site/about/guidelines.xhtml

regulatory body and Research and Ethics Committee prior to participant recruitment. Documents will be stored securely with restricted access for at least 15 years.

Written, informed consent will be obtained from each patient, and an identification number provided. Any published data will not contain personally identifiable data. Study results will be published in a peer-reviewed journal.

Author's contributions

The protocol was devised by Dr Angela Lamarca, Ms Lynne McCallum, Dr Alison Backen and Mr Marc Abraham. Professor Juan W Valle supervised the development of the study protocol and approved the final version. Dr Jorge Barriuso provided independent statistical support. Dr Kate Vaughan coordinated study set-up and supervised the study as project manager. Ms Lindsay Carnie is responsible for patient recruitment and dietetic assessment. Prof Juan W Valle, Dr Richard A Hubner, Dr Mairéad G McNamara and Dr Angela Lamarca, are responsible for identifying and discussing with potentially eligible patients. All authors approved the manuscript.

Peer review was undertaken during protocol development, and the study has been adopted by the National Cancer Research Institute Upper GI Clinical Studies Group (Pancreatic subgroup).

Funding statement

Ms Lindsay Carnie was funded by Pancreatic Cancer UK Fellowship (Clinical Pioneer Award 2015) and Neuroendocrine Cancer UK, formerly known as the NET Patient Foundation. Dr Angela Lamarca has received funding from European Society for Medical Oncology (ESMO) Fellowship Programme, Pancreatic Cancer Research Fund, Spanish Society of Medical Oncology (SEOM) Fellowship Programme, American Society of Clinical Oncology (ASCO) Conquer Cancer Foundation Young Investigator Award and The Christie Charity. Dr Zainul Abedin Kapacee was funded by The Christie

For peer review only - http://bmjopen.bmj.com/site/about/guidelines.xhtml

Charity. Dr Kate Vaughan is funded by Cancer Research UK [Funder reference: C2930/A25234, University of Manchester reference: R120976]

Ethical approval

Ethical approval has been granted by the North West- Greater Manchester East Research Ethics Committee (REC), reference: 17/NW/0597 favourable opinion granted 7th December 2017.

The charity Pancreatic Cancer UK was involved in reviewing the protocol. This manuscript reflects the latest protocol Version 8, approved 21st April, 2020.

IRAS project ID: 194255.

Study sponsor: The Christie NHS Foundation Trust Research and Development Department, Manchester, UK.

For peer review only

References

1 Cade JE, Hanison J. The pancreas. *Anaesth Intensive Care Med* 2017;**18**:527–31. doi:10.1016/j.mpaic.2017.06.021

2 Malvezzi M, Bertuccio P, Levi F, *et al.* European cancer mortality predictions for the year 2014. *Ann Oncol Off J Eur Soc Med Oncol* 2014;**25**:1650–6. doi:10.1093/annonc/mdu138

3 Porta M, Fabregat X, Malats N, *et al.* Exocrine pancreatic cancer: symptoms at presentation and their relation to tumour site and stage. *Clin Transl Oncol Off Publ Fed Span Oncol Soc Natl Cancer Inst Mex* 2005;**7**:189–97. doi:10.1007/bf02712816

4 Hendifar AE, Petzel MQB, Zimmers TA, *et al.* Pancreas Cancer-Associated Weight Loss. *The Oncologist* 2019;**24**:691–701. doi:10.1634/theoncologist.2018-0266

5 Neoptolemos JP, Palmer DH, Ghaneh P, *et al.* Comparison of adjuvant gemcitabine and capecitabine with gemcitabine monotherapy in patients with resected pancreatic cancer (ESPAC-4): a multicentre, open-label, randomised, phase 3 trial. *Lancet Lond Engl* 2017;**389**:1011–24. doi:10.1016/S0140-6736(16)32409-6

6 Oettle H, Post S, Neuhaus P, *et al.* Adjuvant chemotherapy with gemcitabine vs observation in patients undergoing curative-intent resection of pancreatic cancer: a randomized controlled trial. *JAMA* 2007;**297**:267–77. doi:10.1001/jama.297.3.267

7 Conroy T, Hammel P, Hebbar M, *et al.* FOLFIRINOX or Gemcitabine as Adjuvant Therapy for Pancreatic Cancer. *N Engl J Med* 2018;**379**:2395–406. doi:10.1056/NEJMoa1809775

8 Valle JW, Palmer D, Jackson R, *et al.* Optimal duration and timing of adjuvant chemotherapy after definitive surgery for ductal adenocarcinoma of the pancreas: ongoing lessons from the ESPAC-3 study. *J Clin Oncol Off J Am Soc Clin Oncol* 2014;**32**:504–12. doi:10.1200/JCO.2013.50.7657

9 Burris HA, Moore MJ, Andersen J, *et al.* Improvements in survival and clinical benefit with gemcitabine as first-line therapy for patients with advanced pancreas cancer: a randomized trial. *J Clin Oncol Off J Am Soc Clin Oncol* 1997;**15**:2403–13. doi:10.1200/JCO.1997.15.6.2403

10 Von Hoff DD, Ervin T, Arena FP, *et al.* Increased Survival in Pancreatic Cancer with nab-Paclitaxel plus Gemcitabine. *N Engl J Med* 2013;**369**:1691–703. doi:10.1056/NEJMoa1304369

11 Conroy T, Desseigne F, Ychou M, *et al.* FOLFIRINOX versus Gemcitabine for Metastatic Pancreatic Cancer. http://dx.doi.org/10.1056/NEJMoa1011923. 2011. doi:10.1056/NEJMoa1011923

12 McCallum L, Department of Medical Oncology, The Christie NHS Foundation Trust, Manchester M20 4BX, Manchester, United Kingdom, Lamarca A, *et al.* Prevalence of symptomatic pancreatic exocrine insufficiency in patients with pancreatic malignancy: nutritional intervention may improve survival. *Cancer Res Front* 2016;**2**:352–67. doi:10.17980/2016.352

13 Dasari A, Shen C, Halperin D, *et al.* Trends in the Incidence, Prevalence, and Survival Outcomes in Patients With Neuroendocrine Tumors in the United States. *JAMA Oncol* 2017;**3**:1335–42. doi:10.1001/jamaoncol.2017.0589

For peer review only - http://bmjopen.bmj.com/site/about/guidelines.xhtml

14 Akirov A, Larouche V, Alshehri S, *et al.* Treatment Options for Pancreatic Neuroendocrine Tumors. *Cancers* 2019;**11**. doi:10.3390/cancers11060828

15 Clement DS, Tesselaar ME, van Leerdam ME, *et al.* Nutritional and vitamin status in patients with neuroendocrine neoplasms. *World J Gastroenterol* 2019;**25**:1171–84. doi:10.3748/wjg.v25.i10.1171

16 Lindkvist B. Diagnosis and treatment of pancreatic exocrine insufficiency. *World J Gastroenterol* 2013;**19**:7258–66. doi:10.3748/wjg.v19.i42.7258

17 Sikkens ECM, Cahen DL, de Wit J, *et al.* Prospective assessment of the influence of pancreatic cancer resection on exocrine pancreatic function. *Br J Surg* 2014;**101**:109–13. doi:10.1002/bjs.9342

18 Sikkens ECM, Cahen DL, de Wit J, *et al.* A prospective assessment of the natural course of the exocrine pancreatic function in patients with a pancreatic head tumor. *J Clin Gastroenterol* 2014;**48**:e43-46. doi:10.1097/MCG.0b013e31829f56e7

19 Halloran CM, Cox TF, Chauhan S, *et al.* Partial pancreatic resection for pancreatic malignancy is associated with sustained pancreatic exocrine failure and reduced quality of life: a prospective study. *Pancreatol Off J Int Assoc Pancreatol IAP Al* 2011;**11**:535–45. doi:10.1159/000333308

20 Gooden HM, White KJ. Pancreatic cancer and supportive care--pancreatic exocrine insufficiency negatively impacts on quality of life. *Support Care Cancer Off J Multinatl Assoc Support Care Cancer* 2013;**21**:1835–41. doi:10.1007/s00520-013-1729-3

21 Watson L. Exocrine insufficiency and pancreatic enzyme replacement therapy in pancreatic cancer. *Clin Oncol R Coll Radiol G B* 2010;**22**:391. doi:10.1016/j.clon.2010.03.004

22 Bachmann J, Ketterer K, Marsch C, *et al.* Pancreatic cancer related cachexia: influence on metabolism and correlation to weight loss and pulmonary function. *BMC Cancer* 2009;**9**:255. doi:10.1186/1471-2407-9-255

23 Bachmann J, Heiligensetzer M, Krakowski-Roosen H, *et al.* Cachexia worsens prognosis in patients with resectable pancreatic cancer. *J Gastrointest Surg Off J Soc Surg Aliment Tract* 2008;**12**:1193–201. doi:10.1007/s11605-008-0505-z

24 Sikkens ECM, Cahen DL, Kuipers EJ, *et al.* Pancreatic enzyme replacement therapy in chronic pancreatitis. *Best Pract Res Clin Gastroenterol* 2010;**24**:337–47. doi:10.1016/j.bpg.2010.03.006

25 Dumasy V, Delhaye M, Cotton F, *et al.* Fat malabsorption screening in chronic pancreatitis. *Am J Gastroenterol* 2004;**99**:1350–4. doi:10.1111/j.1572-0241.2004.30661.x

26 Burton P, Evans DG, Harper AA, *et al.* A test of pancreatic function in man based on the analysis of duodenal contents after administration of secretin and pancreozymin. *Gut* 1960;**1**:111–24. doi:10.1136/gut.1.2.111

27 Nakamura H, Murakami Y, Uemura K, *et al.* Reduced pancreatic parenchymal thickness indicates exocrine pancreatic insufficiency after pancreatoduodenectomy. *J Surg Res* 2011;**171**:473–8. doi:10.1016/j.jss.2010.03.052

For peer review only

For peer review only - http://bmjopen.bmj.com/site/about/guidelines.xhtml

28 Benini L, Amodio A, Campagnola P, *et al.* Fecal elastase-1 is useful in the detection of steatorrhea in patients with pancreatic diseases but not after pancreatic resection. *Pancreatol Off J Int Assoc Pancreatol IAP Al* 2013;**13**:38–42. doi:10.1016/j.pan.2012.11.307

29 Domínguez-Muñoz JE, Iglesias-García J, Vilariño-Insua M, *et al.* 13C-mixed triglyceride breath test to assess oral enzyme substitution therapy in patients with chronic pancreatitis. *Clin Gastroenterol Hepatol Off Clin Pract J Am Gastroenterol Assoc* 2007;**5**:484–8. doi:10.1016/j.cgh.2007.01.004

30 Lindkvist B, Domínguez-Muñoz JE, Luaces-Regueira M, *et al.* Serum nutritional markers for prediction of pancreatic exocrine insufficiency in chronic pancreatitis. *Pancreatology* 2012;**12**:305–10. doi:10.1016/j.pan.2012.04.006

31 Pancreatric Section, British Society of Gastroenterology, Pancreatic Society of Great Britain and Ireland, Association of Upper Gastrointestinal Surgeons of Great Britain and Ireland, *et al.* Guidelines for the management of patients with pancreatic cancer periampullary and ampullary carcinomas. *Gut* 2005;**54 Suppl 5**:v1-16. doi:10.1136/gut.2004.057059

32 Sabater L, Ausania F, Bakker OJ, *et al.* Evidence-based Guidelines for the Management of Exocrine Pancreatic Insufficiency After Pancreatic Surgery. *Ann Surg* 2016;**264**:949–58. doi:10.1097/SLA.0000000000001732

33 Toouli J, Biankin AV, Oliver MR, *et al.* Management of pancreatic exocrine insufficiency: Australasian Pancreatic Club recommendations. *Med J Aust* 2010;**193**:461–7.

34 Domínguez-Muñoz JE. Chronic pancreatitis and persistent steatorrhea: what is the correct dose of enzymes? *Clin Gastroenterol Hepatol Off Clin Pract J Am Gastroenterol Assoc* 2011;**9**:541–6. doi:10.1016/j.cgh.2011.02.027

35 Domínguez-Muñoz JE, Iglesias-García J, Iglesias-Rey M, *et al.* Optimising the therapy of exocrine pancreatic insufficiency by the association of a proton pump inhibitor to enteric coated pancreatic extracts. *Gut* 2006;**55**:1056–7. doi:10.1136/gut.2006.094912

36 Bean JF, Kiely DK, LaRose S, *et al.* Is stair climb power a clinically relevant measure of leg power impairments in at-risk older adults? *Arch Phys Med Rehabil* 2007;**88**:604–9. doi:10.1016/j.apmr.2007.02.004

37 Roig M, Eng JJ, MacIntyre DL, *et al.* Associations of the Stair Climb Power Test with muscle strength and functional performance in people with chronic obstructive pulmonary disease: a cross-sectional study. *Phys Ther* 2010;**90**:1774–82. doi:10.2522/ptj.20100091

38 Blauwhoff-Buskermolen S, Ruijgrok C, Ostelo RW, *et al.* The assessment of anorexia in patients with cancer: cut-off values for the FAACT-A/CS and the VAS for appetite. *Support Care Cancer Off J Multinatl Assoc Support Care Cancer* 2016;**24**:661–6. doi:10.1007/s00520-015-2826-2

39 Ribaudo JM, Cella D, Hahn EA, *et al.* Re-validation and shortening of the Functional Assessment of Anorexia/Cachexia Therapy (FAACT) questionnaire. *Qual Life Res Int J Qual Life Asp Treat Care Rehabil* 2000;**9**:1137–46. doi:10.1023/a:1016670403148

For peer review only - http://bmjopen.bmj.com/site/about/guidelines.xhtml

40 Walkowiak J, Lisowska A, Przyslawski J, *et al.* Faecal elastase-1 test is superior to faecal lipase test in the assessment of exocrine pancreatic function in cystic fibrosis. *Acta Paediatr Oslo Nor 1992* 2004;**93**:1042–5. doi:10.1111/j.1651-2227.2004.tb02715.x

41 Leeds JS, Hopper AD, Hurlstone DP, *et al.* Is exocrine pancreatic insufficiency in adult coeliac disease a cause of persisting symptoms? *Aliment Pharmacol Ther* 2007;**25**:265–71. doi:10.1111/j.1365-2036.2006.03206.x

42 Barthet M, Lesavre N, Desplats S, *et al.* Frequency and characteristics of pancreatitis in patients with inflammatory bowel disease. *Pancreatol Off J Int Assoc Pancreatol IAP Al* 2006;**6**:464–71. doi:10.1159/000094564

43 Maconi G, Dominici R, Molteni M, *et al.* Prevalence of pancreatic insufficiency in inflammatory bowel diseases. Assessment by fecal elastase-1. *Dig Dis Sci* 2008;**53**:262–70. doi:10.1007/s10620-007-9852-y

44 Leeds JS, Hopper AD, Sidhu R, *et al.* Some patients with irritable bowel syndrome may have exocrine pancreatic insufficiency. *Clin Gastroenterol Hepatol Off Clin Pract J Am Gastroenterol Assoc* 2010;**8**:433–8. doi:10.1016/j.cgh.2009.09.032

45 Hardt PD, Krauss A, Bretz L, *et al.* Pancreatic exocrine function in patients with type 1 and type 2 diabetes mellitus. *Acta Diabetol* 2000;**37**:105–10. doi:10.1007/s005920070011

46 Icks A, Haastert B, Giani G, *et al.* Low fecal elastase-1 in type I diabetes mellitus. *Z Gastroenterol* 2001;**39**:823–30. doi:10.1055/s-2001-17867

47 Rathmann W, Haastert B, Icks A, *et al.* Low faecal elastase 1 concentrations in type 2 diabetes mellitus. *Scand J Gastroenterol* 2001;**36**:1056–61. doi:10.1080/003655201750422657

48 Martin L, Birdsell L, Macdonald N, *et al.* Cancer cachexia in the age of obesity: skeletal muscle depletion is a powerful prognostic factor, independent of body mass index. *J Clin Oncol Off J Am Soc Clin Oncol* 2013;**31**:1539–47. doi:10.1200/JCO.2012.45.2722

49 Common Terminology Criteria for Adverse Events (CTCAE). *HttpswwweortcbeservicesdocctcCTCAE4032010-06-14QuickReference5x7pdf* 2010.

For peer review only

For peer review only - http://bmjopen.bmj.com/site/about/guidelines.xhtml

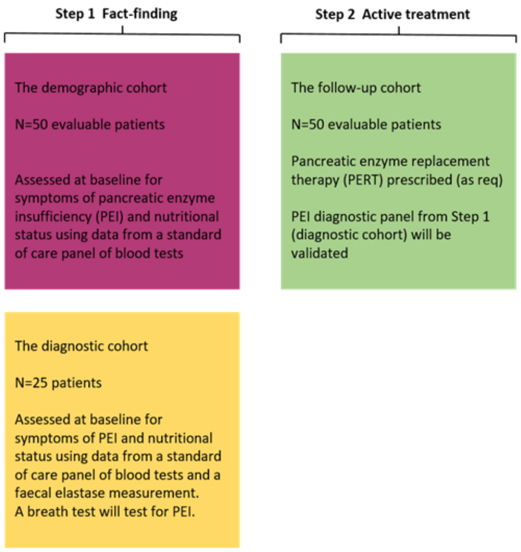

Figure 1: Study design overview

Figure 1: Study design overview

254x190mm (96 x 96 DPI)

	Demographic cohort N = 50	Diagnostic cohort N = 25	Follow up cohort N = 50
**Primary objectives**	**Prospective assessment of PEI prevalence**	**Design and evaluate an optimal PEI diagnostic panel**	**Prospective evaluation of the designed diagnostic panel**
**Secondary objectives**	To determine the prevalence of PEI-related symptoms at first oncological referral.<br>To assess the proportion of patients receiving PERT at the time of oncological referral.<br>To evaluate nutritional status of patients at the time of oncological referral (using data from a panel of 'standard of care' blood tests, weight, Body Mass Index [BMI], Mid-Upper Arm Circumference [MUAC] (reflects both fat mass and fat-free mass), handgrip strength (Measurement of upper body function) and Stair Climb test [SC-test} (to calculate stair climb power)).<br>To evaluate anorexia at baseline by using the FAACT-A/CS (with VAS) (functional Assessment of Anorexia Cachexia Tool (Anorexia Cachexia Scale) with Visual Analogue Scale).	In additional to secondary objectives for demographic cohort;<br><br>To assess the feasibility of performing the PEI breath test and data from a standard of care faecal elastase-1 measurement.<br>To assess, using the "acceptability questionnaire" (developed specifically for this study), the acceptability of these investigations by patients.	Percentage of patients who completed in full the proposed diagnostic panel.<br>Percentage of patients with a positive experience of the diagnostic panel performed ("acceptability questionnaire"), developed specifically for this study.<br>Median change in QoL, FAACT-A/CS (with VAS), BMI, weight, MUAC, handgrip strength and SC-test between baseline and follow-up assessment after 6 weeks, 3 months and 6 months of follow-up.<br>Proportion of patients with PEI -related symptoms at baseline, 6 weeks, 3 months and 6 months from recruitment.<br>Proportion of patients who have a normalisation of the diagnostic panel after 6 weeks, 3 months and 6 months from recruitment.<br>Percentage of patients with good compliance (defined as taking at least 80% of the doses of PERT suggested by dietitian).<br>Percentage of patients who develop any grade PERT-related toxicities.<br>Percentage of patients with a positive experience of the dietetic intervention provided ("feedback questionnaire"), developed specifically for this study.<br>Median OS of the whole population.<br>Percentage of patients starting anti-cancer therapy.<br>Median dose intensity of the received anti-cancer therapy.<br>Correlation between radiological findings and nutritional status measurements.

Figure 2: Study objectives

Figure 2: Study objectives

254x190mm (96 x 96 DPI)

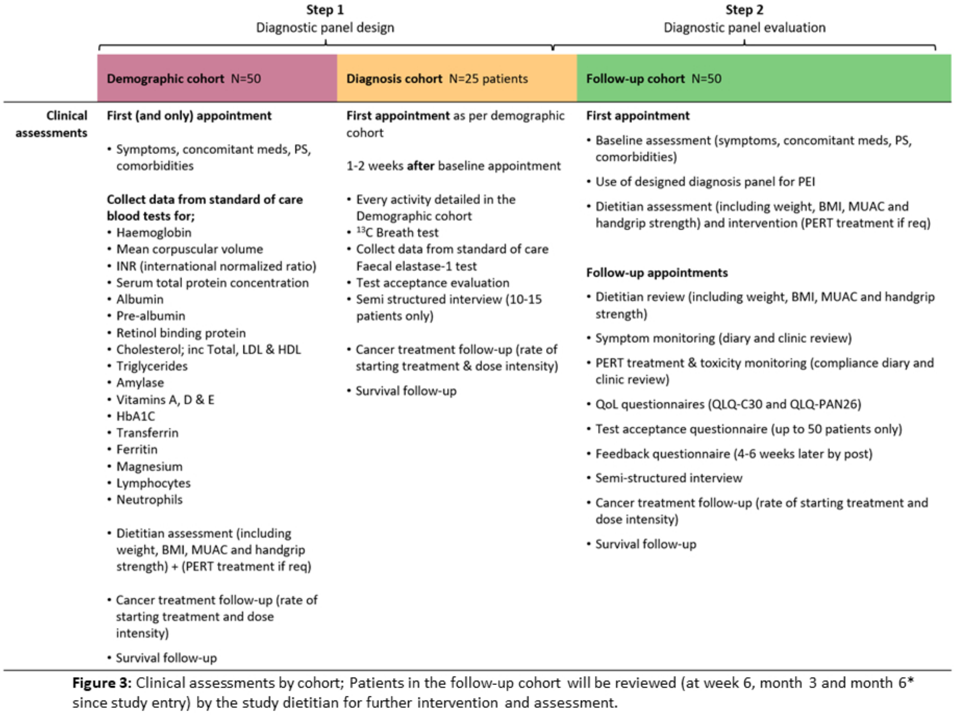

Figure 3: Clinical assessments by cohort; Patients in the follow-up cohort will be reviewed (at week 6, month 3 and month 6* since study entry) by the study dietitian for further intervention and assessment.

254x190mm (96 x 96 DPI)

Prospective observational study of prevalence, assessment and treatment of pancreatic exocrine insufficiency in patients with inoperable pancreatic malignancy (PANcreatic cancer Dietary Assessment - PanDA): a study protocol

Journal:	*BMJ Open*
Manuscript ID	bmjopen-2020-042067.R1
Article Type:	Protocol
Date Submitted by the Author:	11-Oct-2020

Complete List of Authors:	Carnie, Lindsay; The Christie NHS Foundation Trust, Nutrition & Dietetics<br>Lamarca, Angela; The Christie NHS Foundation Trust, Medical Oncology Department; The University of Manchester, Division of Cancer Sciences<br>Vaughan, Kate; The Christie NHS Foundation Trust, Medical Oncology; The University of Manchester, Division of Cancer Sciences<br>Kapacee, Zainul Abedin ; The Christie NHS Foundation Trust, Medical Oncology<br>Mccallum, Lynne; Pancreatic Cancer UK<br>Backen, Alison; The Christie NHS Foundation Trust; The University of Manchester, Division of Cancer Sciences<br>Barriuso, Jorge; The Christie NHS Foundation Trust, Medical Oncology; The University of Manchester, Division of Cancer Sciences<br>McNamara, Mairéad ; The Christie NHS Foundation Trust; The University of Manchester, Division of Cancer Sciences<br>Hubner, R; The Christie NHS Foundation Trust, Medical Oncology; The University of Manchester, Division of Cancer Sciences<br>Abraham†, Marc; The Christie NHS Foundation Trust, Nutrition & Dietetics<br>Valle, Juan; The Christie NHS Foundation Trust, Medical Oncology; The University of Manchester, Division of Cancer Sciences

**Primary Subject Heading**:	Oncology
Secondary Subject Heading:	Nutrition and metabolism
Keywords:	Pancreatic disease < GASTROENTEROLOGY, Gastrointestinal tumours < ONCOLOGY, Nutritional support < ONCOLOGY, NUTRITION & DIETETICS

SCHOLARONE™
Manuscripts

For peer review only - http://bmjopen.bmj.com/site/about/guidelines.xhtml

I, the Submitting Author has the right to grant and does grant on behalf of all authors of the Work (as defined in the below author licence), an exclusive licence and/or a non-exclusive licence for contributions from authors who are: i) UK Crown employees; ii) where BMJ has agreed a CC-BY licence shall apply, and/or iii) in accordance with the terms applicable for US Federal Government officers or employees acting as part of their official duties; on a worldwide, perpetual, irrevocable, royalty-free basis to BMJ Publishing Group Ltd ("BMJ") its licensees and where the relevant Journal is co-owned by BMJ to the co-owners of the Journal, to publish the Work in this journal and any other BMJ products and to exploit all rights, as set out in our licence.

The Submitting Author accepts and understands that any supply made under these terms is made by BMJ to the Submitting Author unless you are acting as an employee on behalf of your employer or a postgraduate student of an affiliated institution which is paying any applicable article publishing charge ("APC") for Open Access articles. Where the Submitting Author wishes to make the Work available on an Open Access basis (and intends to pay the relevant APC), the terms of reuse of such Open Access shall be governed by a Creative Commons licence – details of these licences and which Creative Commons licence will apply to this Work are set out in our licence referred to above.

Other than as permitted in any relevant BMJ Author's Self Archiving Policies, I confirm this Work has not been accepted for publication elsewhere, is not being considered for publication elsewhere and does not duplicate material already published. I confirm all authors consent to publication of this Work and authorise the granting of this licence.

For peer review only

Contents

Title Page 3

Abstract 4

Strengths and limitations of the study 5

Introduction 6

The pancreas 6

Pancreatic cancer | The importance of being fit for treatment 6

Pancreatic exocrine insufficiency | Causing malnutrition in patients with pancreatic cancer 7

Diagnosis of PEI in patients with pancreatic malignancy 8

Treatment of PEI and its impact on Quality of Life and survival 9

Aim 10

Study design 10

Study objectives and patient eligibility 10

Demographic cohort 10

Diagnostic cohort 12

Follow-up cohort 12

Clinical assessments 13

Demographic cohort 14

Screening and Visit 1 14

Follow-up visits 14

Diagnostic cohort 15

Screening and Visit 1 15

For peer review only

Follow-up visits 15

Follow-up cohort 15

Screening and Visit 1 16

Visit 2 (within two weeks) 16

Week 4-6 from study entry 16

Follow-up visits 16

Statistical analysis 17

Sample size 17

Study end-points 18

Demographic cohort; 18

Diagnostic cohort; 18

Follow-up cohort; 18

Data analysis 18

Ethics and dissemination 19

Patient and Public Involvement 20

Author's contributions 20

Funding statement 21

Competing interests statement 21

Ethical approval 22

References 23

For peer review only

For peer review only - http://bmjopen.bmj.com/site/about/guidelines.xhtml

Title Page

Prospective observational study of prevalence, assessment and treatment of pancreatic exocrine insufficiency in patients with inoperable pancreatic malignancy (PANcreatic cancer Dietary Assessment - PanDA): a study protocol

List of authors

Lindsay Carnie [(1)], Angela Lamarca [(2, 3)], Kate Vaughan [(2, 3)], Zainul Abedin Kapacee [(2)], Lynne McCallum [(4)], Alison Backen [(2,3)], Jorge Barriuso[(2,3)], Mairéad G McNamara[(2, 3)], Richard A Hubner[(2, 3)], Marc Abraham [(1)], Juan W Valle [(2, 3)]

List of affiliations

1. Nutrition & Dietetics, The Christie NHS Foundation Trust, Manchester, UK
2. Medical Oncology Department, The Christie NHS Foundation Trust, Manchester, UK
3. Division of Cancer Sciences, University of Manchester, UK
4. Pancreatic Cancer UK, London, UK

Corresponding author: Prof Juan W Valle, Medical Oncology Department, The Christie NHS Foundation Trust, Wilmslow Road, Manchester, M20 4BX, United Kingdom. E-mail: juan.valle@christie.nhs.uk

Key words: pancreatic ductal adenocarcinoma, pancreatic neuroendocrine tumours, dietitian, malnutrition, pancreatic exocrine insufficiency, pancreatic enzyme replacement therapy, breath test, faecal elastase

Word Count: 3733 words (excluding title page, abstract, references, figures and tables)

For peer review only - http://bmjopen.bmj.com/site/about/guidelines.xhtml

Abstract

Introduction

Pancreatic exocrine insufficiency (PEI) in patients with pancreatic malignancy is well documented in the literature and is known to negatively impact on overall survival and quality of life. A lack of consensus opinion remains on the optimal diagnostic test that can be adapted for use in a clinical setting for this cohort of patients. This study aims to better understand the prevalence of PEI and the most suitable diagnostic techniques in patients with advanced pancreatic malignancy.

Methods and analysis

This prospective observational study, will be carried out in patients with pancreatic malignancy (including adenocarcinoma and neuroendocrine neoplasms). Consecutive patients with inoperable pancreatic malignancy referred for consideration of first-line chemotherapy will be considered for eligibility. The study comprises of three cohorts: demographic cohort (primary objective to prospectively investigate the prevalence of PEI in patients with inoperable pancreatic malignancy) ; sample size 50, diagnostic cohort (primary objective to design and evaluate an optimal diagnostic panel to detect PEI in patients with inoperable pancreatic malignancy); sample size 25 and follow-up cohort (primary objective to prospectively evaluate the proposed PEI diagnostic panel in a cohort of patients with inoperable pancreatic malignancy); sample size 50. The following is a summary of the protocol and methodology.

Ethics and dissemination

Full ethical approval has been granted by the North West Greater Manchester East Research and Ethics Committee, reference: 17/NW/0597. This manuscript reflects the latest protocol v.8 approved 21st April, 2020. Findings will be disseminated by presentation in national/international conferences, publication in peer review journals and distribution via patient advocate groups.

For peer review only

Trial registration number

IRAS project ID: 194255.

Clinicaltrials.gov ID: NCT03616431

Strengths and limitations of the study

- This prospective study is a first-of-its-kind aiming to better define PEI, its diagnosis and treatment, in patients with inoperable pancreatic malignancy
- Findings from the demographic cohort will define the prevalence of PEI in patients with inoperable pancreatic malignancy, while the diagnostic cohort will define the most suitable test/panel to define PEI in this setting.
- Results will be validated in the follow-up cohort, including impact on patients' quality of life
- Due to the nature of PEI and the fact that PEI treatment is considered standard-of-care, this is a non-randomised study in which all patients will be exposed to PEI treatment (if required) and dietitian input
- We expect limited statistical power and capacity to assess impact of PEI-related intervention on quality of life and patient outcome derived from characteristics of the study population, study design and limited sample size.

For peer review only - http://bmjopen.bmj.com/site/about/guidelines.xhtml

Introduction

The pancreas

The pancreas has two main functions; producing enzymes to digest protein, fat and carbohydrates into smaller molecules that the body can absorb, and producing hormones that regulate metabolism (including the regulation of blood sugar levels (insulin and glucagon) and global regulation of other hormones). [1]

Pancreatic cancer | The importance of being fit for treatment

Pancreatic cancer (adenocarcinoma) is known to have a poor prognosis with a very low cure rate; most patients diagnosed will die of the disease. In 2014, around 41,000 pancreatic cancer-related deaths occurred in Europe.[2]

The physical location of the tumour can prevent the digestive regulatory functions of the pancreas, causing the systemic symptoms that the majority of patients present with. Symptoms include anorexia (83%), asthenia (86%) and weight loss (85%). [3] Symptoms can impact on Quality of Life (QoL), nutritional status and performance status (PS), which subsequently may preclude active treatment options such as chemotherapy.[4]

Only approximately 20% of patients are suitable for surgery at diagnosis; these patients undergo pancreatic resection followed by adjuvant chemotherapy with fluoropyrimidine-based or gemcitabine-based treatment.[5] [6] [7] A good nutritional status, prior to adjuvant chemotherapy increases the likelihood of a patient completing chemotherapy, which in turn impacts on survival. [8]

Most patients (80%) present with advanced disease and are unsuitable for surgery. Instead they will receive palliative chemotherapy, aiming to improve QoL and prolong overall survival (OS). Single-agent gemcitabine has long been considered standard of care in patients with a poorer performance

For peer review only

status, providing a median OS of 6 months. [9] Recent chemotherapy combinations show improved results, reaching a median OS of 8.5 months (nab-paclitaxel/gemcitabine), [10] and 11.1 months (FOLFIRINOX; a 5-fluorouracil, oxaliplatin and irinotecan combination). [11]

A retrospective analysis of patients with advanced pancreatic cancer referred to The Christie NHS Foundation Trust found around 40% were not fit for active treatment due to poor baseline PS as per The Eastern Cooperative Oncology Group – PS (ECOG-PS) definition. [12]

The scenario for patients diagnosed with pancreatic neuroendocrine tumours (PanNETs) differs significantly. Prognosis is measured in term of years, with an estimated median OS of 3.6 years [13] and multiple options of systemic therapy are currently available.[14] The prevalence of PanNETs is rare, with an estimated incidence of 0.8 per 100,000. [13] Whilst the prognosis of these patients is better, this longer survival time means that identified and minimising the impact of nutritional deficiencies and issues is of particular importance. [15]

Pancreatic exocrine insufficiency | Causing malnutrition in patients with pancreatic cancer

Pancreatic exocrine insufficiency (PEI) is defined as "a reduction in pancreatic enzyme activity in the intestinal lumen to a level below the threshold required to maintain normal digestion".[16]

A high prevalence of PEI has been described in patients with resected (>80%) [17] or advanced-disease (92%) [18] in prospective series, and this negatively impacts on QoL. [19] Different mechanisms have been postulated for the development of PEI, including loss of functioning pancreatic parenchyma (by tumour infiltration or resection or concurrent/prior pancreatitis) and/or pancreatic duct obstruction. PEI, leading to maldigestion, steatorrhoea and malnutrition, has been proposed as a leading cause for the high number of patients with pancreatic malignancy being unfit for active treatment. [20]

For peer review only

Whilst healthcare professionals seem aware of the importance of diagnosing and treating PEI in patients after pancreatic resection, it is often overlooked in patients with advanced disease. This under-recognition and under-treatment of PEI in patients with advanced disease is an ongoing issue, requiring urgent action. [21]

Weight loss is a poor prognostic factor in patients with both resectable and advanced pancreatic malignancy. [22,23] However, little published information exists on the extent of nutritionally-mediated weight loss, how this relates to the cancer, and how much could be mitigated with pro-active pancreatic enzyme replacement therapy (PERT).

Diagnosis of PEI in patients with pancreatic malignancy

Waiting for symptom development, including steatorrhoea (defined as excess fat in faeces that appears when 90% of pancreatic function is lost) delays the diagnosis of PEI and negatively impacts on nutrition and QoL. [24] Early assessment of exocrine function is fundamental, and should be considered in all patients diagnosed with pancreatic disorders, including cancer. [25]

Diagnosing PEI in patients with pancreatic malignancy can be difficult, and a lack of consensus remains for the optimal assessment method. Whilst three-day faecal fat quantification is 'gold-standard' for diagnosing PEI, its use in clinical practice is challenging. [16] The secretin test is invasive and has potential for clinical complications, reducing its appeal. [26] Measurable reduction of pancreatic parenchymal thickness in imaging correlates with changes assessed using a ^{13}C-mixed triglyceride breath test (^{13}C-MTBT), with good sensitivity and specificity after pancreatic resection. [27] This has become the new 'standard', replacing the three-day faecal fat test. The use of current diagnostic techniques such as faecal elastase-1 (FE-1), [28] (postulated to be more useful in patients who have not undergone resection), the ^{13}C-MTBT [29] and a nutritional panel of blood-based markers warrant further investigation to clarify their use. [30]

For peer review only - http://bmjopen.bmj.com/site/about/guidelines.xhtml

In summary | The optimal diagnostic method for PEI in patients with pancreatic malignancy remains undefined; ^{13}C-MTBT is considered 'gold-standard' but is challenging to apply in daily clinical settings. This study aims to design the most appropriate and least-invasive diagnostic panel, with ^{13}C-MTBT as the comparator for patients diagnosed with pancreatic malignancy (including both adenocarcinoma and neuroendocrine tumours).

Treatment of PEI and its impact on Quality of Life and survival

Guidelines for the management of PEI exist,[31–33] and two publications support using high-dose PERT to mimic the physiological situation, to normalise nutritional status.[29,34] Using a proton pump inhibitor to increase gastric pH, enhancing the efficacy of PERT (by reducing gastric acid-induced enzymatic degradation) in selected patients has also been demonstrated. [35]

At The Christie NHS Foundation Trust, 183 patients with pancreatic malignancy were retrospectively analysed and it was demonstrated that patients receiving nutritional intervention (PERT, nutritional supplements or dietitian support) seemed to receive more chemotherapy and had a longer OS [10.2 months (95%CI 7.5-13.3) vs 6.9 months (95%CI 5.5-9.9); HR 0.6 (95%CI 0.4-0.9); p-value 0.015], when adjusted for other variables in the multivariable analysis (type of pancreatic cancer, stage at diagnosis, ECOG-PS and chemotherapy treatment)]. [12] This study also confirmed that PEI is under-recognised and under-treated in patients with advanced disease. Since this was a retrospective study, it is subject to selection and survival bias. Therefore, whilst results are encouraging, prospective studies are required to evaluate the impact of dietetic intervention (including PERT) on QoL, exposure to anti-cancer treatment, symptom control and outcome.

In summary | Dietetic intervention, early diagnosis and management of PEI could impact patients' OS. This study aims to prospectively assess the impact of such interventions in patients with pancreatic malignancy.

For peer review only - http://bmjopen.bmj.com/site/about/guidelines.xhtml

Aim

This prospective observational study aims to evaluate;

- The prevalence of PEI in patients with pancreatic ductal adenocarcinoma and PanNETs (hence force termed pancreatic malignancy).
- The most appropriate diagnostic strategy.
- The impact of adequate diagnosis and treatment of PEI on patient treatment and outcomes.

Study design

The study will be conducted in two steps, as summarised in **Figure 1**.

Step-1 | A prospective cross-sectional assessment of the prevalence of PEI-related symptoms in patients with pancreatic malignancy (this will be termed 'the **Demographic cohort**'). A separate cohort of patients will be tested to elucidate the most efficient diagnostic panel for PEI in pancreatic malignancy (this will be termed 'the **Diagnostic cohort**').

Step-2 | A prospective longitudinal validation of the diagnostic panel designed and tested in Step-1 and evaluation of dietitian intervention (including PERT) and its impact on weight loss, symptom evolution, chemotherapy dose-intensity, QoL and OS (this will be termed 'the **Follow-up cohort**').

Study objectives and patient eligibility

A summary of study objectives are provided in **Figure 2**.

Demographic cohort

The primary objective is to prospectively investigate the prevalence of PEI in patients with inoperable pancreatic malignancy. Prevalence will be determined by the presence of symptoms

For peer review only

deemed in-keeping with PEI by the research dietitian; alongside the absence of another causes for symptoms or standard diagnostic techniques (FE-1).

Secondary objectives include, at baseline oncological appointment;

- To assess the proportion of patients receiving PERT
- To evaluate nutritional status (using a panel of blood tests (including nutritional parameters), weight, Body Mass Index [BMI], Mid-Upper Arm Circumference [MUAC] (reflects both fat mass and fat-free mass), handgrip strength (measures upper body function) and Stair Climb test [SC-test] (to calculate stair climb power[36,37]))
- To evaluate anorexia, using the Functional Assessment of Anorexia/Cachexia Therapy questionnaire (FAACT–A/CS) and Visual Analogue Scale (VAS) [38,39]

Eligible patients for the demographic cohort are those who have biopsy-proven or clinically-suspected (by specialist multidisciplinary team (MDT) meeting) inoperable (locally-advanced or metastatic) pancreatic ductal adenocarcinoma (and variants) or PanNET. There is no minimal time-frame for patients to have been diagnosed with cancer. Patients must be ≥18 years and able to provide written, informed consent and are being considered for first-line chemotherapy. Patients with PanNET may have received previous systemic treatment, but cannot be on active treatment.

Patients are deemed ineligible if they have had previous gastric, duodenal or pancreatic resections, if they have an intolerance/aversion to pork-containing products for religious or personal reasons. Additionally, patients are ineligible if they have comorbidities that increase the probability of PEI, including but not limited to: chronic pancreatitis, [25] cystic fibrosis,[40] coeliac disease,[41] inflammatory bowel disease,[42,43] diarrhoea-dominant irritable bowel syndrome,[44] diabetes diagnosed > 5 years ago. [45–47]

For peer review only - http://bmjopen.bmj.com/site/about/guidelines.xhtml

Diagnostic cohort

The primary objective is to design and evaluate an optimal diagnostic panel to detect PEI in patients with inoperable pancreatic malignancy.

In addition to the secondary objectives of the Demographic cohort;

- To assess the feasibility and acceptability (using a specifically designed “Acceptability Questionnaire”) of the ^{13}C-MTBT and the FE-1 test

Eligible patients for the diagnostic cohort are those who fulfil the eligibility criteria for the demographic cohort. In addition, patients with potentially operable disease but who have not undergone surgery for whatever reason (i.e. co-morbidities) would be eligible if all other eligibility criteria are met. Additionally, patients diagnosed with adenocarcinoma (and variants) will be allowed to have received previous systemic treatment but will require to be off active treatment for a minimum of 3 months to be included in this cohort.

In addition to the exclusion criteria for the demographic cohort, patients must not be allergic to metoclopramide, a prokinetic used in the ^{13}C-MTBT.

Follow-up cohort

The primary objective is to prospectively evaluate the proposed PEI diagnostic panel in a cohort of patients with inoperable pancreatic malignancy.

Secondary objectives include;

- To evaluate the feasibility of applying the designed diagnostic panel in clinical practice and to assess the patient acceptability of these investigations

For peer review only - http://bmjopen.bmj.com/site/about/guidelines.xhtml

- To quantify changes to nutritional status (BMI, weight, MUAC, handgrip and SC-test), evaluate symptoms, and extent of diagnostic panel normalisation, anorexia (FAACT–A/CS (with VAS) and QoL at 6 weeks, 3 months and 6 months after recruitment and dietetic input
- To evaluate PERT compliance and toxicity
- To assess patient perceptions of the dietetic care provided
- To evaluate the impact of dietetic intervention on OS, anti-cancer therapy starting rate & anti-cancer therapy dose intensity
- Exploratory radiological surrogates (i.e. intra-abdominal fat or psoas muscle measurements) from standard of care Computed Tomography (CT) scans and its correlation with nutritional assessment may be investigated
- To evaluate the median dose intensity of the received anti-cancer therapy and the correlation between radiological findings and nutritional status measurements [48]

Eligible patients for the follow-up cohort are those fulfilling the eligibility criteria for the diagnostic cohort and if further follow-up at The Christie NHS Foundation Trust is planned.

Clinical assessments

Consecutive patients with inoperable pancreatic malignancy referred for consideration of first-line systemic therapy will be considered for eligibility. Eligible patients will be provided with verbal and written study information and given sufficient time to consider participation.

Clinical assessments will be undertaken as per **Figure 3**. All consenting patients will undergo a prospective assessment of nutritional status, will be screened for PEI and will receive tailored advice by the research dietitian in established oncology clinics.

For peer review only - http://bmjopen.bmj.com/site/about/guidelines.xhtml

Demographic cohort

Screening and Visit 1

At baseline, patients will be screened against inclusion criteria. Written, informed consent must be granted before patients are registered. If appropriate, the baseline assessment can be performed on the same day as screening.

Assessments undertaken are as follows (**Figure 3**):

- Physical examination; vital signs, height, weight, BMI, MUAC, handgrip strength, SC-test and FAACT-A/CS (with VAS)
- Baseline symptoms (PEI-related) and graded as per Common Terminology Criteria for Adverse Events version 4.03 (CTCAE v 4.03) [49]
- PEI-relevant concomitant medications
- ECOG-PS
- Blood collection for nutritional panel
- Dietitian assessment and counselling (PERT will be commenced, if required)
- PERT treatment and toxicity assessment (if patient on PERT)

Follow-up visits

No follow-up visits will be required. Beyond study participation, dietetic input will be provided as per standard of care outside the context of this study. Information on subsequent chemotherapy treatment (starting rate and dose intensity) and survival outcomes will be collected.

For peer review only

For peer review only - http://bmjopen.bmj.com/site/about/guidelines.xhtml

Diagnostic cohort

Screening and Visit 1

The baseline assessment will be completed as per the “Demographic cohort”.

Visit 2 (prior to starting systemic treatments)

The following assessments will be performed:

- Weight
- FE-1 test (container provided at baseline visit and returned at visit 2)
- ^{13}C-MTBT (takes around six hours to complete, including administering bread spread with ^{13}C butter and subsequent collection of the patient’s breath in small breath bags at timed intervals, which will be analysed for ^{13}C quantity)
- Acceptability questionnaire for FE-1 test and ^{13}C-MTBT (all patients) to provide opinions on the burden that these extra tests may add

Follow-up visits

No follow-up visits will be required. Patients attending clinic for further follow-up/treatment, will have further dietetic input, as required, outside of the context of this study. Information on the subsequent chemotherapy (starting rate and dose intensity) and survival outcomes will be collected.

Follow-up cohort

Prior to opening recruitment to this cohort, data from the Demographic and Diagnostic cohorts will be analysed, which will dictate the most informative diagnostic panel devised to be used in the Follow-up cohort.

For peer review only - http://bmjopen.bmj.com/site/about/guidelines.xhtml

Screening and Visit 1

Screening will be completed as per the Demographic and Diagnostic cohorts.

In addition, further assessments (**Figure 3**) include:

- QoL questionnaires (QLQ-C30 (all patients) and either QLQ-PAN26 (pancreatic ductal adenocarcinoma) or QLQ NET-21 (PanNET))
- A symptom and PERT diary for data collection will be provided

Visit 2 (within two weeks)

- Designed PEI diagnostic panel (from all the potential combinations of FE-1 test, symptom assessment, nutritional assessment [weight, BMI, MUAC, handgrip strength, SC-test, FAACT–A/CS (with VAS) and nutritional blood panel]), as per findings from the Diagnostic cohort)
- "Acceptability Questionnaire" regarding the burden that this diagnostic panel added

Week 4-6 from study entry

- "Feedback questionnaire" regarding perception of dietetic input (all patients) (posted to the patient and returned using a provided stamped-addressed envelope)

Follow-up visits

At six weeks, three months and six months after recruitment;

- Weight, BMI, MUAC, handgrip strength, SC-test and FAACT-A/CS (with VAS)
- Physical examination (symptom directed), including vital signs (if appropriate)
- ECOG-PS
- Dietitian assessment and counselling, nutrition support advice (diet, nutritional supplements, etc.) and PERT, as required

For peer review only

- PERT treatment review and toxicity assessment (if taking PERT)
- Symptom and PERT diary collection
- QoL questionnaires repeated as per visit 1
- Survival and chemotherapy treatment monitoring (retrospectively)

Patients attending clinic for further follow-up/treatment will be provided with dietetic input, as required, outside of the context of this study.

Statistical analysis

Sample size

No formal sample size calculation was performed. Instead, a realistic estimation of the number of patients possible to recruit was made using established referral rates and the length of time this study will recruit for. A high drop-out rate was expected due to the poor outcomes of patients with pancreatic cancer. Therefore, sufficient patients will be recruited to ensure the planned number of 'evaluable patients' for each cohort is reached. These are defined as;

Demographic cohort: up to 50 eligible patients completing the assessment required in "Visit 1 "

Diagnostic cohort: up to 25 eligible patients willing (at time of consent) to complete breath test and other cohort-dependent examinations

Follow-up cohort: up to 50 eligible patients completing assessments up to and including "Follow-up visit week 6"

Handgrip strength measurements contribute to the statistical calculation; In order to gather supporting data, a non-selected sample of 12 patients with pancreatic ductal adenocarcinoma performed the handgrip test as per standard of care assessment at The Christie (mean percentile was 75; standard deviation of 16). Using these data, the Follow-up cohort (50 patients with handgrip

For peer review only - http://bmjopen.bmj.com/site/about/guidelines.xhtml

For peer review only

assessment at baseline and at 6 weeks) will have a power of 0.75 to show an improvement from 75 to 82 (seven point improvement). This is assuming an alpha error of 0.15 and same standard deviation in both baseline and 6-week assessment handgrip results (standard deviation of 16). This is supporting evidence that the sample size for this study will be able to provide meaningful and robust results.

Study end-points

The primary end-points of the three cohorts are;

Demographic cohort;

- Proportion of patients with symptoms/findings in keeping with a PEI diagnosis

Diagnostic cohort;

- Odds ratio for prediction of diagnosis of PEI (measured by ^{13}C-MTBT) of the most accurate diagnostic panel (designed from all potential combinations of FE-1 test, symptom assessment, nutritional assessment [weight, BMI, MUAC, handgrip strength, SC-test, FAACT–A/CS (with VAS) and nutritional blood panel])

Follow-up cohort;

- Rate of PEI diagnosis according to the designed diagnostic panel (diagnostic cohort)

Data analysis

Frequency tables for all categorical variables, arranged by category, will be produced for comparison. Continuous variables (age, weight, BMI, MUAC, handgrip strength, SC-test and FAACT–A/CS (with VAS) will be presented, using the median and range (minimum, maximum) or mean (variance), depending on whether data distribution appears symmetrical.

For peer review only - http://bmjopen.bmj.com/site/about/guidelines.xhtml

For exploratory analyses, means will be compared using either Student T test (if parametric validity conditions are fulfilled) or non-parametric Wilcoxon-Mann-Whitney tests. Proportions will be compared using either Chi-squared statistics or Fisher exact test, as appropriate. Toxicity data will be tabulated. The worst toxicity grade over all cycles according to the CTCAE v 4.03 [49] will be reported.

Median survival will be calculated using the Kaplan-Meier estimator technique. Median OS will be displayed with the 95% confidence interval. For comparison of survival curves, Log-rank test will be applied. Multivariable analyses will also be performed (Cox Regression).

Analysis of data collected from the Demographic and Diagnostic cohorts will be undertaken to devise the optimal diagnostic panel, using ^{13}C-MTBT as a reference to diagnose PEI. Results from the breath test will be reported as a dichotomised variable (normal or abnormal).

Logistic regression will be performed, aiming to choose the most informative, but simplest panel of tests, to predict PEI as the ^{13}C-MTBT has done.

Individually measured blood parameters, together with other calculated scores (such as, but not limited to the "prognostic nutritional index" (combining lymphocytes and albumin)) will be included in such analysis, if required.

Further analysis upon the completion of the Follow-up cohort will evaluate the panel's accuracy and acceptability for use in clinical practice.

Ethics and dissemination

Full ethical approval has been granted by the North West Greater Manchester East Research and Ethics Committee, reference: 17/NW/0597. This manuscript reflects the latest protocol v.8 approved 21st April, 2020.

For peer review only - http://bmjopen.bmj.com/site/about/guidelines.xhtml

The study will be conducted according to the principles of Good Clinical Practice (GCP), General Data Protection Regulation (GDPR) and data protection Act 2018 for Health and Care Research. The sponsor and study team will ensure approval of the study protocol, participant information sheets, consent forms, letters to General Practitioners and supporting documents by the appropriate regulatory body and Research and Ethics Committee prior to participant recruitment. Documents will be stored securely with restricted access for at least 15 years.

Written, informed consent will be obtained from each patient, and an identification number provided. Any published data will not contain personally identifiable data. Findings will be disseminated by presentation in national/international conferences, publication in peer review journals and distribution *via* patient advocate groups.

Patient and Public Involvement

Patient advocate groups [Pancreatic Cancer UK and Neuroendocrine Cancer UK (formerly known as the NET Patient Foundation)] were involved in the development of this study protocol. Results will be disseminated to patients *via* these advocate groups once results are available.

Author's contributions

The protocol was devised by Dr Angela Lamarca, Ms Lynne McCallum, Dr Alison Backen and Mr Marc Abraham. Professor Juan W Valle supervised the development of the study protocol and approved the final version. Dr Jorge Barriuso provided independent statistical support. Dr Kate Vaughan coordinated study set-up and supervised the study as project manager. Ms Lindsay Carnie is responsible for patient recruitment and dietetic assessment. Prof Juan W Valle, Dr Richard A Hubner, Dr Mairéad G McNamara, Dr Zainul Abedin Kapacee and Dr Angela Lamarca, are responsible for identifying and discussing with potentially eligible patients. All authors approved the manuscript.

For peer review only

For peer review only - http://bmjopen.bmj.com/site/about/guidelines.xhtml

Peer review was undertaken during protocol development, and the study has been adopted by the National Cancer Research Institute Upper GI Clinical Studies Group (Pancreatic subgroup).

Funding statement

Ms Lindsay Carnie was funded by Pancreatic Cancer UK Fellowship (Clinical Pioneer Award 2015) and Neuroendocrine Cancer UK, formerly known as the NET Patient Foundation. Dr Kate Vaughan is funded by Cancer Research UK [Funder reference: C2930/A25234, University of Manchester reference: R120976]

Competing interests statement

Ms. Carnie reports grants from Pancreatic Cancer UK and NET patient foundation, during the conduct of the study; Travel and educational support from Mylan and IPSEN, outside the submitted work; Dr. Lamarca reports grants and personal fees from: Travel and educational support from Ipsen, Pfizer, Bayer, AAA, SirtEx, Novartis, Mylan and Delcath. Speaker honoraria from Merck, Pfizer, Ipsen, Incyte and AAA. Advisory honoraria from EISAI, Nutricia Ipsen, QED and Roche. Member of the Knowledge Network and NETConnect Initiatives funded by Ipsen. All outside the submitted work; Dr. Kapacee reports educational and travel support from EISAI, outside the submitted work; Dr. McNamara reports grants from NuCana, grants from Servier, grants from Ipsen, other from Novartis, outside the submitted work; Dr. Hubner reports personal fees from Ipsen, Mylan, Celgene and PrimeOncology, outside the submitted work; Mr Abraham reports Travel and educational support from Mylan, outside the submitted work; Dr. Valle reports personal fees from AstraZeneca, Debiopharm, Delcath Systems, Genoscience Pharma, Imaging Equipment Limited, Incyte, Ipsen, Keocyt, Merck, Mundipharma EDO, Novartis, PCI Biotech, Pieris Pharmaceuticals, QED, Wren Laboratories and Agios; grants, personal fees and non-financial support from NuCana, personal fees and non-financial support from Pfizer, grants and personal fees from Servier, outside the submitted work; Dr Vaughan, Ms McCallum, Dr Backen and Dr Burriuso have nothing to declare.

For peer review only

Acknowledgement

Authors would like to state that patient advocate groups [Pancreatic Cancer UK and Neuroendocrine Cancer UK (formerly known as the NET Patient Foundation)] were involved in the development of this study protocol. Dr Angela Lamarca has received funding from European Society for Medical Oncology (ESMO) Fellowship Programme, Pancreatic Cancer Research Fund, Spanish Society of Medical Oncology (SEOM) Fellowship Programme, American Society of Clinical Oncology (ASCO) Conquer Cancer Foundation Young Investigator Award and The Christie Charity. Dr Zainul Abedin Kapacee was funded by The Christie Charity.

Ethical approval

Ethical approval has been granted by the North West- Greater Manchester East Research Ethics Committee (REC), reference: 17/NW/0597 favourable opinion granted 7th December 2017.

The charity Pancreatic Cancer UK was involved in reviewing the protocol. This manuscript reflects the latest protocol Version 8, approved 21st April, 2020.

IRAS project ID: 194255.

Study sponsor: The Christie NHS Foundation Trust Research and Development Department, Manchester, UK.

For peer review only

References

1 Cade JE, Hanison J. The pancreas. *Anaesth Intensive Care Med* 2017;**18**:527–31. doi:10.1016/j.mpaic.2017.06.021

2 Malvezzi M, Bertuccio P, Levi F, *et al.* European cancer mortality predictions for the year 2014. *Ann Oncol Off J Eur Soc Med Oncol* 2014;**25**:1650–6. doi:10.1093/annonc/mdu138

3 Porta M, Fabregat X, Malats N, *et al.* Exocrine pancreatic cancer: symptoms at presentation and their relation to tumour site and stage. *Clin Transl Oncol Off Publ Fed Span Oncol Soc Natl Cancer Inst Mex* 2005;**7**:189–97. doi:10.1007/bf02712816

4 Hendifar AE, Petzel MQB, Zimmers TA, *et al.* Pancreas Cancer-Associated Weight Loss. *The Oncologist* 2019;**24**:691–701. doi:10.1634/theoncologist.2018-0266

5 Neoptolemos JP, Palmer DH, Ghaneh P, *et al.* Comparison of adjuvant gemcitabine and capecitabine with gemcitabine monotherapy in patients with resected pancreatic cancer (ESPAC-4): a multicentre, open-label, randomised, phase 3 trial. *Lancet Lond Engl* 2017;**389**:1011–24. doi:10.1016/S0140-6736(16)32409-6

6 Oettle H, Post S, Neuhaus P, *et al.* Adjuvant chemotherapy with gemcitabine vs observation in patients undergoing curative-intent resection of pancreatic cancer: a randomized controlled trial. *JAMA* 2007;**297**:267–77. doi:10.1001/jama.297.3.267

7 Conroy T, Hammel P, Hebbar M, *et al.* FOLFIRINOX or Gemcitabine as Adjuvant Therapy for Pancreatic Cancer. *N Engl J Med* 2018;**379**:2395–406. doi:10.1056/NEJMoa1809775

8 Valle JW, Palmer D, Jackson R, *et al.* Optimal duration and timing of adjuvant chemotherapy after definitive surgery for ductal adenocarcinoma of the pancreas: ongoing lessons from the ESPAC-3 study. *J Clin Oncol Off J Am Soc Clin Oncol* 2014;**32**:504–12. doi:10.1200/JCO.2013.50.7657

9 Burris HA, Moore MJ, Andersen J, *et al.* Improvements in survival and clinical benefit with gemcitabine as first-line therapy for patients with advanced pancreas cancer: a randomized trial. *J Clin Oncol Off J Am Soc Clin Oncol* 1997;**15**:2403–13. doi:10.1200/JCO.1997.15.6.2403

10 Von Hoff DD, Ervin T, Arena FP, *et al.* Increased Survival in Pancreatic Cancer with nab-Paclitaxel plus Gemcitabine. *N Engl J Med* 2013;**369**:1691–703. doi:10.1056/NEJMoa1304369

11 Conroy T, Desseigne F, Ychou M, *et al.* FOLFIRINOX versus Gemcitabine for Metastatic Pancreatic Cancer. http://dx.doi.org/10.1056/NEJMoa1011923. 2011. doi:10.1056/NEJMoa1011923

12 McCallum L, Department of Medical Oncology, The Christie NHS Foundation Trust, Manchester M20 4BX, Manchester, United Kingdom, Lamarca A, *et al.* Prevalence of symptomatic pancreatic exocrine insufficiency in patients with pancreatic malignancy: nutritional intervention may improve survival. *Cancer Res Front* 2016;**2**:352–67. doi:10.17980/2016.352

13 Dasari A, Shen C, Halperin D, *et al.* Trends in the Incidence, Prevalence, and Survival Outcomes in Patients With Neuroendocrine Tumors in the United States. *JAMA Oncol* 2017;**3**:1335–42. doi:10.1001/jamaoncol.2017.0589

For peer review only - http://bmjopen.bmj.com/site/about/guidelines.xhtml

14 Akirov A, Larouche V, Alshehri S, *et al.* Treatment Options for Pancreatic Neuroendocrine Tumors. *Cancers* 2019;**11**. doi:10.3390/cancers11060828

15 Clement DS, Tesselaar ME, van Leerdam ME, *et al.* Nutritional and vitamin status in patients with neuroendocrine neoplasms. *World J Gastroenterol* 2019;**25**:1171–84. doi:10.3748/wjg.v25.i10.1171

16 Lindkvist B. Diagnosis and treatment of pancreatic exocrine insufficiency. *World J Gastroenterol* 2013;**19**:7258–66. doi:10.3748/wjg.v19.i42.7258

17 Sikkens ECM, Cahen DL, de Wit J, *et al.* Prospective assessment of the influence of pancreatic cancer resection on exocrine pancreatic function. *Br J Surg* 2014;**101**:109–13. doi:10.1002/bjs.9342

18 Sikkens ECM, Cahen DL, de Wit J, *et al.* A prospective assessment of the natural course of the exocrine pancreatic function in patients with a pancreatic head tumor. *J Clin Gastroenterol* 2014;**48**:e43-46. doi:10.1097/MCG.0b013e31829f56e7

19 Halloran CM, Cox TF, Chauhan S, *et al.* Partial pancreatic resection for pancreatic malignancy is associated with sustained pancreatic exocrine failure and reduced quality of life: a prospective study. *Pancreatol Off J Int Assoc Pancreatol IAP Al* 2011;**11**:535–45. doi:10.1159/000333308

20 Gooden HM, White KJ. Pancreatic cancer and supportive care--pancreatic exocrine insufficiency negatively impacts on quality of life. *Support Care Cancer Off J Multinatl Assoc Support Care Cancer* 2013;**21**:1835–41. doi:10.1007/s00520-013-1729-3

21 Watson L. Exocrine insufficiency and pancreatic enzyme replacement therapy in pancreatic cancer. *Clin Oncol R Coll Radiol G B* 2010;**22**:391. doi:10.1016/j.clon.2010.03.004

22 Bachmann J, Ketterer K, Marsch C, *et al.* Pancreatic cancer related cachexia: influence on metabolism and correlation to weight loss and pulmonary function. *BMC Cancer* 2009;**9**:255. doi:10.1186/1471-2407-9-255

23 Bachmann J, Heiligensetzer M, Krakowski-Roosen H, *et al.* Cachexia worsens prognosis in patients with resectable pancreatic cancer. *J Gastrointest Surg Off J Soc Surg Aliment Tract* 2008;**12**:1193–201. doi:10.1007/s11605-008-0505-z

24 Sikkens ECM, Cahen DL, Kuipers EJ, *et al.* Pancreatic enzyme replacement therapy in chronic pancreatitis. *Best Pract Res Clin Gastroenterol* 2010;**24**:337–47. doi:10.1016/j.bpg.2010.03.006

25 Dumasy V, Delhaye M, Cotton F, *et al.* Fat malabsorption screening in chronic pancreatitis. *Am J Gastroenterol* 2004;**99**:1350–4. doi:10.1111/j.1572-0241.2004.30661.x

26 Burton P, Evans DG, Harper AA, *et al.* A test of pancreatic function in man based on the analysis of duodenal contents after administration of secretin and pancreozymin. *Gut* 1960;**1**:111–24. doi:10.1136/gut.1.2.111

27 Nakamura H, Murakami Y, Uemura K, *et al.* Reduced pancreatic parenchymal thickness indicates exocrine pancreatic insufficiency after pancreatoduodenectomy. *J Surg Res* 2011;**171**:473–8. doi:10.1016/j.jss.2010.03.052

For peer review only - http://bmjopen.bmj.com/site/about/guidelines.xhtml

28 Benini L, Amodio A, Campagnola P, *et al.* Fecal elastase-1 is useful in the detection of steatorrhea in patients with pancreatic diseases but not after pancreatic resection. *Pancreatol Off J Int Assoc Pancreatol IAP Al* 2013;**13**:38–42. doi:10.1016/j.pan.2012.11.307

29 Domínguez-Muñoz JE, Iglesias-García J, Vilariño-Insua M, *et al.* 13C-mixed triglyceride breath test to assess oral enzyme substitution therapy in patients with chronic pancreatitis. *Clin Gastroenterol Hepatol Off Clin Pract J Am Gastroenterol Assoc* 2007;**5**:484–8. doi:10.1016/j.cgh.2007.01.004

30 Lindkvist B, Domínguez-Muñoz JE, Luaces-Regueira M, *et al.* Serum nutritional markers for prediction of pancreatic exocrine insufficiency in chronic pancreatitis. *Pancreatology* 2012;**12**:305–10. doi:10.1016/j.pan.2012.04.006

31 Pancreatric Section, British Society of Gastroenterology, Pancreatic Society of Great Britain and Ireland, Association of Upper Gastrointestinal Surgeons of Great Britain and Ireland, *et al.* Guidelines for the management of patients with pancreatic cancer periampullary and ampullary carcinomas. *Gut* 2005;**54 Suppl 5**:v1-16. doi:10.1136/gut.2004.057059

32 Sabater L, Ausania F, Bakker OJ, *et al.* Evidence-based Guidelines for the Management of Exocrine Pancreatic Insufficiency After Pancreatic Surgery. *Ann Surg* 2016;**264**:949–58. doi:10.1097/SLA.0000000000001732

33 Toouli J, Biankin AV, Oliver MR, *et al.* Management of pancreatic exocrine insufficiency: Australasian Pancreatic Club recommendations. *Med J Aust* 2010;**193**:461–7.

34 Domínguez-Muñoz JE. Chronic pancreatitis and persistent steatorrhea: what is the correct dose of enzymes? *Clin Gastroenterol Hepatol Off Clin Pract J Am Gastroenterol Assoc* 2011;**9**:541–6. doi:10.1016/j.cgh.2011.02.027

35 Domínguez-Muñoz JE, Iglesias-García J, Iglesias-Rey M, *et al.* Optimising the therapy of exocrine pancreatic insufficiency by the association of a proton pump inhibitor to enteric coated pancreatic extracts. *Gut* 2006;**55**:1056–7. doi:10.1136/gut.2006.094912

36 Bean JF, Kiely DK, LaRose S, *et al.* Is stair climb power a clinically relevant measure of leg power impairments in at-risk older adults? *Arch Phys Med Rehabil* 2007;**88**:604–9. doi:10.1016/j.apmr.2007.02.004

37 Roig M, Eng JJ, MacIntyre DL, *et al.* Associations of the Stair Climb Power Test with muscle strength and functional performance in people with chronic obstructive pulmonary disease: a cross-sectional study. *Phys Ther* 2010;**90**:1774–82. doi:10.2522/ptj.20100091

38 Blauwhoff-Buskermolen S, Ruijgrok C, Ostelo RW, *et al.* The assessment of anorexia in patients with cancer: cut-off values for the FAACT-A/CS and the VAS for appetite. *Support Care Cancer Off J Multinatl Assoc Support Care Cancer* 2016;**24**:661–6. doi:10.1007/s00520-015-2826-2

39 Ribaudo JM, Cella D, Hahn EA, *et al.* Re-validation and shortening of the Functional Assessment of Anorexia/Cachexia Therapy (FAACT) questionnaire. *Qual Life Res Int J Qual Life Asp Treat Care Rehabil* 2000;**9**:1137–46. doi:10.1023/a:1016670403148

For peer review only

For peer review only - http://bmjopen.bmj.com/site/about/guidelines.xhtml

40 Walkowiak J, Lisowska A, Przyslawski J, *et al.* Faecal elastase-1 test is superior to faecal lipase test in the assessment of exocrine pancreatic function in cystic fibrosis. *Acta Paediatr Oslo Nor 1992* 2004;**93**:1042–5. doi:10.1111/j.1651-2227.2004.tb02715.x

41 Leeds JS, Hopper AD, Hurlstone DP, *et al.* Is exocrine pancreatic insufficiency in adult coeliac disease a cause of persisting symptoms? *Aliment Pharmacol Ther* 2007;**25**:265–71. doi:10.1111/j.1365-2036.2006.03206.x

42 Barthet M, Lesavre N, Desplats S, *et al.* Frequency and characteristics of pancreatitis in patients with inflammatory bowel disease. *Pancreatol Off J Int Assoc Pancreatol IAP Al* 2006;**6**:464–71. doi:10.1159/000094564

43 Maconi G, Dominici R, Molteni M, *et al.* Prevalence of pancreatic insufficiency in inflammatory bowel diseases. Assessment by fecal elastase-1. *Dig Dis Sci* 2008;**53**:262–70. doi:10.1007/s10620-007-9852-y

44 Leeds JS, Hopper AD, Sidhu R, *et al.* Some patients with irritable bowel syndrome may have exocrine pancreatic insufficiency. *Clin Gastroenterol Hepatol Off Clin Pract J Am Gastroenterol Assoc* 2010;**8**:433–8. doi:10.1016/j.cgh.2009.09.032

45 Hardt PD, Krauss A, Bretz L, *et al.* Pancreatic exocrine function in patients with type 1 and type 2 diabetes mellitus. *Acta Diabetol* 2000;**37**:105–10. doi:10.1007/s005920070011

46 Icks A, Haastert B, Giani G, *et al.* Low fecal elastase-1 in type I diabetes mellitus. *Z Gastroenterol* 2001;**39**:823–30. doi:10.1055/s-2001-17867

47 Rathmann W, Haastert B, Icks A, *et al.* Low faecal elastase 1 concentrations in type 2 diabetes mellitus. *Scand J Gastroenterol* 2001;**36**:1056–61. doi:10.1080/003655201750422657

48 Martin L, Birdsell L, Macdonald N, *et al.* Cancer cachexia in the age of obesity: skeletal muscle depletion is a powerful prognostic factor, independent of body mass index. *J Clin Oncol Off J Am Soc Clin Oncol* 2013;**31**:1539–47. doi:10.1200/JCO.2012.45.2722

49 Common Terminology Criteria for Adverse Events (CTCAE). *HttpswwweortcbeservicesdocctcCTCAE4032010-06-14QuickReference5x7pdf* 2010.

For peer review only

For peer review only - http://bmjopen.bmj.com/site/about/guidelines.xhtml

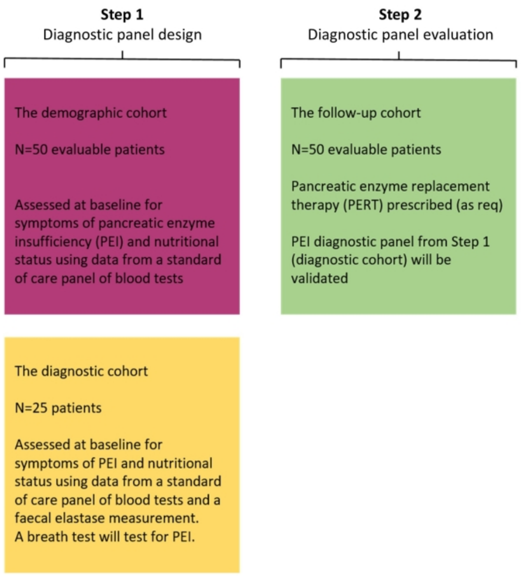

Figure 1: Study design overview

Figure 1

254x190mm (300 x 300 DPI)

	Demographic cohort N = 50	Diagnostic cohort N = 25	Follow up cohort N = 50
**Primary objectives**	**Prospective assessment of PEI prevalence**	**Design and evaluate an optimal PEI diagnostic panel**	**Prospective evaluation of the designed diagnostic panel**
**Secondary objectives**	To determine the prevalence of PEI-related symptoms at first oncological referral.<br>To assess the proportion of patients receiving PERT at the time of oncological referral.<br>To evaluate nutritional status of patients at the time of oncological referral (using data from a panel of 'standard of care' blood tests, weight, Body Mass Index [BMI], Mid-Upper Arm Circumference [MUAC] (reflects both fat mass and fat-free mass), handgrip strength (Measurement of upper body function) and Stair Climb test [SC-test] (to calculate stair climb power)).<br>To evaluate anorexia at baseline by using the FAACT-A/CS (with VAS) (functional Assessment of Anorexia Cachexia Tool (Anorexia Cachexia Scale) with Visual Analogue Scale).	In additional to secondary objectives for demographic cohort;<br><br>To assess the feasibility of performing the PEI breath test and data from a standard of care faecal elastase-1 measurement.<br>To assess, using the "acceptability questionnaire" (developed specifically for this study), the acceptability of these investigations by patients.	Percentage of patients who completed in full the proposed diagnostic panel.<br>Percentage of patients with a positive experience of the diagnostic panel performed ("acceptability questionnaire"), developed specifically for this study.<br>Median change in QoL, FAACT-A/CS (with VAS), BMI, weight, MUAC, handgrip strength and SC-test between baseline and follow-up assessment after 6 weeks, 3 months and 6 months of follow-up.<br>Proportion of patients with PEI -related symptoms at baseline, 6 weeks, 3 months and 6 months from recruitment.<br>Proportion of patients who have a normalisation of the diagnostic panel after 6 weeks, 3 months and 6 months from recruitment.<br>Percentage of patients with good compliance (defined as taking at least 80% of the doses of PERT suggested by dietitian).<br>Percentage of patients who develop any grade PERT-related toxicities.<br>Percentage of patients with a positive experience of the dietetic intervention provided ("feedback questionnaire"), developed specifically for this study.<br>Median OS of the whole population.<br>Percentage of patients starting anti-cancer therapy.<br>Median dose intensity of the received anti-cancer therapy.<br>Correlation between radiological findings and nutritional status measurements.

Figure 2: Study objectives

Figure 2

254x190mm (300 x 300 DPI)

	Step 1<br>Diagnostic panel design		Step 2<br>Diagnostic panel evaluation
	**Demographic cohort** N=50	**Diagnosis cohort** N=25 patients	**Follow-up cohort** N=50
**Clinical assessments**	**First (and only) appointment**<br>• Symptoms, concomitant meds, PS, comorbidities<br>**Collect data from standard of care blood tests for;**<br>• Haemoglobin<br>• Mean corpuscular volume<br>• INR (international normalized ratio)<br>• Serum total protein concentration<br>• Albumin<br>• Pre-albumin<br>• Retinol binding protein<br>• Cholesterol; inc Total, LDL & HDL<br>• Triglycerides<br>• Amylase<br>• Vitamins A, D & E<br>• HbA1C<br>• Transferrin<br>• Ferritin<br>• Magnesium<br>• Lymphocytes<br>• Neutrophils<br>• Dietitian assessment (including weight, BMI, MUAC and handgrip strength) + (PERT treatment if req)<br>• Cancer treatment follow-up (rate of starting treatment and dose intensity)<br>• Survival follow-up	**First appointment** as per demographic cohort<br>1-2 weeks **after** baseline appointment<br>• Every activity detailed in the Demographic cohort<br>• ^{13}C Breath test<br>• Collect data from standard of care Faecal elastase-1 test<br>• Test acceptance evaluation<br>• Semi structured interview (10-15 patients only)<br>• Cancer treatment follow-up (rate of starting treatment & dose intensity)<br>• Survival follow-up	**First appointment**<br>• Baseline assessment (symptoms, concomitant meds, PS, comorbidities)<br>• Use of designed diagnosis panel for PEI<br>• Dietitian assessment (including weight, BMI, MUAC and handgrip strength) and intervention (PERT treatment if req)<br>**Follow-up appointments**<br>• Dietitian review (including weight, BMI, MUAC and handgrip strength)<br>• Symptom monitoring (diary and clinic review)<br>• PERT treatment & toxicity monitoring (compliance diary and clinic review)<br>• QoL questionnaires (QLQ-C30 and QLQ-PAN26)<br>• Test acceptance questionnaire (up to 50 patients only)<br>• Feedback questionnaire (4-6 weeks later by post)<br>• Semi-structured interview<br>• Cancer treatment follow-up (rate of starting treatment and dose intensity)<br>• Survival follow-up

Figure 3: Clinical assessments by cohort; Patients in the follow-up cohort will be reviewed (at week 6, month 3 and month 6* since study entry) by the study dietitian for further intervention and assessment.

Figure 3

254x190mm (300 x 300 DPI)
